# Comparative safety and effectiveness of dabigatran vs. rivaroxaban and apixaban in patients with non-valvular atrial fibrillation: a retrospective study from a large healthcare system

**DOI:** 10.1093/ehjcvp/pvy044

**Published:** 2018-11-30

**Authors:** Todd C Villines, Azhar Ahmad, Michaela Petrini, Wenbo Tang, Amber Evans, Toni Rush, David Thompson, Kelly Oh, Eric Schwartzman

**Affiliations:** 1 Department of Medicine, Cardiology Service, Walter Reed National Military Medical Center, 8901 Wisconsin Avenue, Rockville, MD, USA; 2 Boehringer Ingelheim (Malaysia) Sdn. Bhd. Wisma UOA Damansara II, No 6 Jalan Changkat Semantan, Damansara Height, Kuala Lumpur, Malaysia; 3 Boehringer Ingelheim Pharmaceuticals, Inc., 900 Ridgebury Road Ridgefield, CT, USA; 4 Health ResearchTx, LLC, 5 Neshamy Interplex, Suite 206, Trevose, PA, USA; 5 Syneos Health, 470 Atlantic Ave, Boston, MA, USA; 6 Naval Medical Center Portsmouth, Portsmouth, VA, USA

**Keywords:** Apixaban, Dabigatran, Propensity score matching, Rivaroxaban, US Department of Defense Military Health System

## Abstract

**Aims:**

We used the US Department of Defense Military Health System database to compare the safety and effectiveness of direct oral anticoagulants (DOACs) in patients with non-valvular atrial fibrillation (NVAF) initiating dabigatran vs. rivaroxaban or apixaban.

**Methods and results:**

Two cohorts of adults with NVAF, newly initiated on standard-dose DOAC, were identified based on clinical approval dates: July 2011–June 2016 for dabigatran (150 mg b.i.d.) or rivaroxaban (20 mg QD) and January 2013–June 2016 for dabigatran (150 mg b.i.d.) or apixaban (5 mg b.i.d.). Propensity score matching (1:1) identified two well-balanced cohorts (dabigatran vs. rivaroxaban *n* = 12 763 per treatment group; dabigatran vs. apixaban *n* = 4802 per treatment group). In both cohorts, baseline characteristics and follow-up duration were similar between treatment groups. Patients newly initiating dabigatran had significantly lower risk of major bleeding vs. rivaroxaban [2.08% vs. 2.53%; hazard ratio (HR) 0.82, 95% confidence interval (CI) 0.70–0.97; *P *=* *0.018], while stroke risk was similar (0.60% vs. 0.78%; HR 0.77, 95% CI 0.57–1.04; *P *=* *0.084). The dabigatran vs. apixaban cohort analysis found no differences in risk of major bleeding (1.60% vs. 1.21%; HR 1.37, 95% CI 0.97–1.94; *P *=* *0.070) or stroke (0.44% vs. 0.35%; HR 1.26, 95% CI 0.66–2.39; *P *=* *0.489).

**Conclusion:**

Among NVAF patients newly initiated on standard-dose DOAC therapy in this study, dabigatran was associated with significantly lower major bleeding risk vs. rivaroxaban, and no significant difference in stroke risk. For dabigatran vs. apixaban, the reduced sample size limited the ability to draw definitive conclusions.

## Introduction

Since 2010, direct oral anticoagulants (DOACs) have been available for clinical use in the US for the prevention of thromboembolic stroke and related disorders. Based on large, randomized controlled trials (RCTs) comparing DOACs with warfarin in patients with non-valvular atrial fibrillation (NVAF),^1–4^ current treatment guidelines recommend them as safe and efficacious alternatives to oral anticoagulation with vitamin K antagonists/warfarin.^5^ Despite this, concerns remained about the safety of these agents when applied to a broader clinical practice population, typically with multiple morbidities. Large-scale ‘real-world’ studies comparing DOACs with warfarin have served to reassure healthcare providers that the results of RCTs generally translated to routine clinical care.^6^^,^^7^

A similar approach has been used to compare DOACs, again because of a lack of ‘head-to-head’ RCTs.^8^^,^^9^ For example, Medicare data were used by Graham *et al*.^10^ to study outcomes in elderly, propensity score matched (PSM) patients with NVAF who initiated treatment with dabigatran or rivaroxaban. The investigators concluded that treatment with standard-dose rivaroxaban was associated with statistically significant increases in intracranial haemorrhage and major extracranial bleeding, including major gastrointestinal (GI) bleeding, compared with standard-dose dabigatran. However, the mean follow-up in this study was less than 4 months (108 days and 111 days for the dabigatran and rivaroxaban groups, respectively). Additional studies with a longer follow-up, and involving a greater diversity of patients in real-world clinical settings, would add to the available body of evidence.

The purpose of the present study was to compare the safety and effectiveness of dabigatran, rivaroxaban, and apixaban in a large-scale, real-world cohort of patients with NVAF, who were newly initiated on standard doses.

## Methods

### Data source

The Department of Defense (DoD) Military Health System is a large-scale, comprehensive system with more than 9 million beneficiaries who generally have long-term coverage and extended treatment follow-up in comparison with most patients in commercial insurance plans.^11^^,^^12^ The DoD claims database has previously been used to study oral anticoagulation with dabigatran or warfarin in PSM patients (*n* = 12 793 for both groups). Dabigatran-treated patients had lower rates of stroke, major intracranial bleeding, urogenital bleeding, and other bleeding, as well as fewer myocardial infarctions (MIs) and deaths than warfarin-treated patients. While rates of major bleeding and major GI bleeding were similar in both groups, major lower GI bleeding events were more frequent in the dabigatran-treated patients.^7^

### Study design

This retrospective study compared outcomes in two cohorts of DoD patients with NVAF who were newly initiating DOAC therapy: a dabigatran vs. rivaroxaban cohort and a dabigatran vs. apixaban cohort. To improve comparability and minimize potential bias, comparisons were only made following the approval dates of both compared medications (1 July 2011 for the dabigatran vs. rivaroxaban cohort, 28 December 2011 for the dabigatran vs. apixaban cohort). Although rivaroxaban was approved for stroke prevention in atrial fibrillation (SPAF) on 4 November 2011, the venous thromboembolism approval date for rivaroxaban was used for this analysis. It was confirmed that no one receiving rivaroxaban prior to SPAF was included in the study based on stringent inclusion/exclusion criteria of no other DOAC alternative indication usage allowed. At the time of the analysis, data were available to 30 June 2016.

The index date (baseline) for each patient was defined as the date of their first claim of a DOAC prescription. The pre-index period (lookback period) was the 12 months before the first DOAC prescription claim, during which all patients were to have a NVAF diagnosis and be oral anticoagulation treatment-naïve (defined as having no claim for any oral anticoagulant in the pre-index period). Including the pre-index period, the time frames were 1 July 2010 to 30 June 2016 for the dabigatran vs. rivaroxaban cohort, and 28 December 2011 to 30 June 2016 for the dabigatran vs. apixaban cohort.

Patient follow-up began the day after the DOAC index date, and ended on the earliest occurrence of either (i) discontinuation of the index DOAC exposure (index exposure was considered discontinued if there was a treatment gap longer than the 30-day allowable gap specified from the end of the calculated days supplied), (ii) switching to a different anticoagulant, (iii) a change in index DOAC dosing, (iv) disenrolment, or (v) death. If a patient discontinued the index DOAC or switched to a different anticoagulation therapy, the outcomes assessment did not continue beyond the date of discontinuation or the switch (i.e. there was no latency period). All outcomes were studied using on-treatment analyses rather than initial treatment carried forward analyses.

### Protection of human subjects

This study was reviewed by the Naval Medical Center Portsmouth Institutional Review Board, and conducted in compliance with applicable federal and state laws, including the Health Insurance Portability and Accountability Act of 1996 (HIPAA). Informed consent was waived due to the retrospective nature of the study. All patient data were fully de-identified in compliance with HIPAA regulations, to ensure adherence to the Privacy Rule and to safeguard patient confidentiality. Each patient was assigned a unique, random 15-digit identifier used to link data collected through the retrospective query of the Military Health System Data Repository. A DoD clinical epidemiologist certified the data de-identification, and the study Project Manager maintained a master log.

### Patients

Study subjects were treatment-naïve patients with NVAF, who then had ≥1 prescription claim for dabigatran, rivaroxaban, or apixaban during the patient identification period. Patients were treated according to the recommended standard dosing regimen for each DOAC (apixaban, 5 mg b.i.d.; dabigatran, 150 mg b.i.d.; rivaroxaban, 20 mg QD). Patients receiving edoxaban were not included in this analysis as edoxaban represents a very small fraction (∼0.5%) of the total market for oral anticoagulants in the US.^13^

Included patients were aged ≥18 years on the index date, had ≥12 months of continuous eligibility prior to the index date, and had ≥1 diagnosis code of atrial fibrillation (AF), defined as ICD-9-CM diagnosis of 427.31 or ICD-10-CM diagnosis of I48.0, I48.1, I48.2, and I48.91 on the index date or during the pre-index period. Key exclusion criteria included any claim suggesting transient AF in the 3-month pre-index period, any claim suggesting that the patient had ‘valvular’ AF in the pre-index period, or any instance of cardiac surgery, pericarditis, or myocarditis. A complete list of codes for exclusion diagnoses and procedures are given in Supplementary data online, *Tables S1 and S2*.

**Table 1:** Baseline characteristics for the two cohorts, after propensity score matching

	Dabigatran vs. rivaroxaban		Dabigatran vs. apixaban	
	Dabigatran (*n* = 12 763)	Rivaroxaban (*n* = 12 763)	Dabigatran (*n* = 4802)	Apixaban (*n* = 4802)
Age (years)				
Mean (SD)	70.9 (10.0)	70.9 (10.1)	70.2 (10.2)	70.2 (10.0)
Median (range)	72 (19–85)	72 (18–85)	71 (19–85)	71 (19–85)
Age category (years)				
<65	2864 (22.4)	2722 (21.3)	1171 (24.4)	1137 (23.7)
65–74	4710 (36.9)	4837 (37.9)	1846 (38.4)	1888 (39.3)
75–84	4310 (33.8)	4429 (34.7)	1501 (31.3)	1509 (31.4)
≥85	879 (6.9)	775 (6.1)	284 (5.9)	268 (5.6)
Gender				
Male	7902 (61.9)	7839 (61.4)	3028 (63.1)	3039 (63.3)
Female	4861 (38.1)	4924 (38.6)	1774 (36.9)	1763 (36.7)
Medical history				
Hypertension	9566 (75.0)	9577 (75.0)	3508 (73.1)	3495 (72.8)
Diabetes	3555 (27.9)	3523 (27.6)	1344 (28.0)	1333 (27.8)
Prior stroke (all types)	989 (7.7)	979 (7.7)	338 (7.0)	334 (7.0)
Transient ischaemic attack	618 (4.8)	595 (4.7)	209 (4.4)	197 (4.1)
Congestive heart failure	1802 (14.1)	1797 (14.1)	669 (13.9)	673 (14.0)
Renal disease	1853 (14.5)	1807 (14.2)	733 (15.3)	724 (15.1)
Risk scores, mean (SD)				
Charlson comorbidity index	4.27 (2.41)	4.26 (2.40)	4.17 (2.45)	4.18 (2.44)
CHADS_2_ stroke risk score	1.77 (1.22)	1.77 (1.23)	1.70 (1.21)	1.69 (1.20)
CHA_2_DS_2_-VASc stroke risk score	3.10 (1.66)	3.10 (1.63)	2.98 (1.65)	2.97 (1.61)
HAS-BLED bleed risk score^a^	2.3 (1.2)	2.3 (1.2)	2.3 (1.2)	2.3 (1.2)

Values are expressed as *n* (%) except where indicated.

INR, international normalized ratio; SD, standard deviation.

a Based on the modified HAS-BLED risk score with a maximum score of 8 because INR data/information were not available for all patients in DoD data.

**Table 2:** Primary outcome event rates and hazard ratios for the two cohorts, after propensity score matching (on-treatment analysis)

	Dabigatran vs. rivaroxaban			Dabigatran vs. apixaban		
	Dabigatran (*n* = 12 763)	Rivaroxaban (*n* = 12 763)	*P*-value	Dabigatran (*n* = 4802)	Apixaban (*n* = 4802)	*P*-value
Stroke (overall)^a^						
Patients with event, *n* (%)	77 (0.60)	100 (0.78)		21 (0.44)	17 (0.35)	
Event rate per 100 person-years (95% CI)	0.52 (0.41–0.66)	0.69 (0.56–0.84)		0.46 (0.28–0.70)	0.36 (0.21–0.58)	
Hazard ratio (95% CI)	0.77 (0.57–1.04)		0.084	1.26 (0.66–2.39)		0.489
Major bleeding (overall)^b^						
Patients with event *n* (%)	266 (2.08)	323 (2.53)		77 (1.60)	58 (1.21)	
Event rate per 100 person-years (95% CI)	1.82 (1.60–2.05)	2.24 (2.00–2.49)		1.69 (1.33–2.11)	1.24 (0.94–1.60)	
Hazard ratio (95% CI)	0.82 (0.70–0.97)		0.018	1.37 (0.97–1.94)		0.070

CI, confidence interval.

a Stroke includes ischaemic and haemorrhagic stroke.

b Major bleeding includes haemorrhagic stroke, major intracranial bleeding, and major extracranial bleeding.

### Outcomes

For the purposes of the analysis we defined primary, secondary, and additional outcomes. The primary efficacy outcome was stroke (including haemorrhagic or ischaemic) and the primary safety outcome was major bleeding (including haemorrhagic stroke, major intracranial bleeding, or major extracranial bleeding). Secondary outcomes included type of major bleeding (intracranial, extracranial, GI, or other), type of stroke (ischaemic or haemorrhagic), transient ischaemic attack, and all-cause mortality. Additional outcomes were MIs, and venous thromboembolic events, presenting as either deep vein thrombosis or pulmonary embolism. All outcome ICD-9 and corresponding ICD-10 codes are listed in Supplementary data online, *Table S3*.

### Study size

Formal sample size calculations were not undertaken in the study protocol, but the minimum effect size that could be detected with sufficient power was estimated from the expected number of patients in the DoD database. The primary safety outcome of major bleeding was used for power assessments, assuming an annual event rate of 3.1% for the dabigatran patients and mean follow-up duration of 0.82 years for both groups, based on previously published data.^7^ Based on these hypotheses, 11 682 PSM patients per group would be sufficient to detect a relative difference in the hazard of major bleeding of 22% with 80% power. For 12 763 and 4802 PSM patients per group, the power to detect a 22% difference would be 85% and 30%, respectively.

### Statistical methods

All safety and effectiveness outcomes were assessed separately in each of the two cohorts (dabigatran vs. rivaroxaban and dabigatran vs. apixaban). Baseline characteristics for the two cohorts of DOAC-treated patients were summarized using standard descriptive statistics. Logistic regression analysis was performed to derive propensity scores, reflecting the estimated likelihood of each patient being dispensed dabigatran based on baseline demographics and clinical characteristics. The baseline variables included in the models were proposed *a priori* based on medical knowledge (variables are listed in Supplementary data online, *Tables S4 and S5*); the index year was not included in the propensity score model. We used nearest neighbour 1:1 matching of dabigatran to rivaroxaban and dabigatran to apixaban patients within a caliper of 0.20 of the standard deviation of the propensity scores.

To examine the effectiveness of propensity score matching in balancing baseline characteristics within the matched cohorts, standardized differences (STD) were calculated for the variables included in the propensity score model. The matched cohorts were considered balanced if the absolute value of the STD was ≤10%. A full listing of all the variables included in the models can be found in Supplementary data online, *Tables S4 and S5*.

Outcome event incidence rates and 95% confidence intervals (CI) were calculated using a person-time approach for each outcome among each treatment group. Incidence rates were based on the total number of patients in each treatment group who had the outcome during follow-up divided by the total person-years at risk in the cohort.

Kaplan–Meier cumulative incidence plots were generated to characterize risk over time. Cox proportional-hazards regression was used to evaluate the association between DOAC treatment and time-to-event. Statistical significance was assessed at the two-sided alpha level of 0.05. All statistical analyses were performed using SAS version 9.3 (SAS Institute, Cary, NC, USA).

## Results

### Dabigatran vs. rivaroxaban

**Figure 1:**
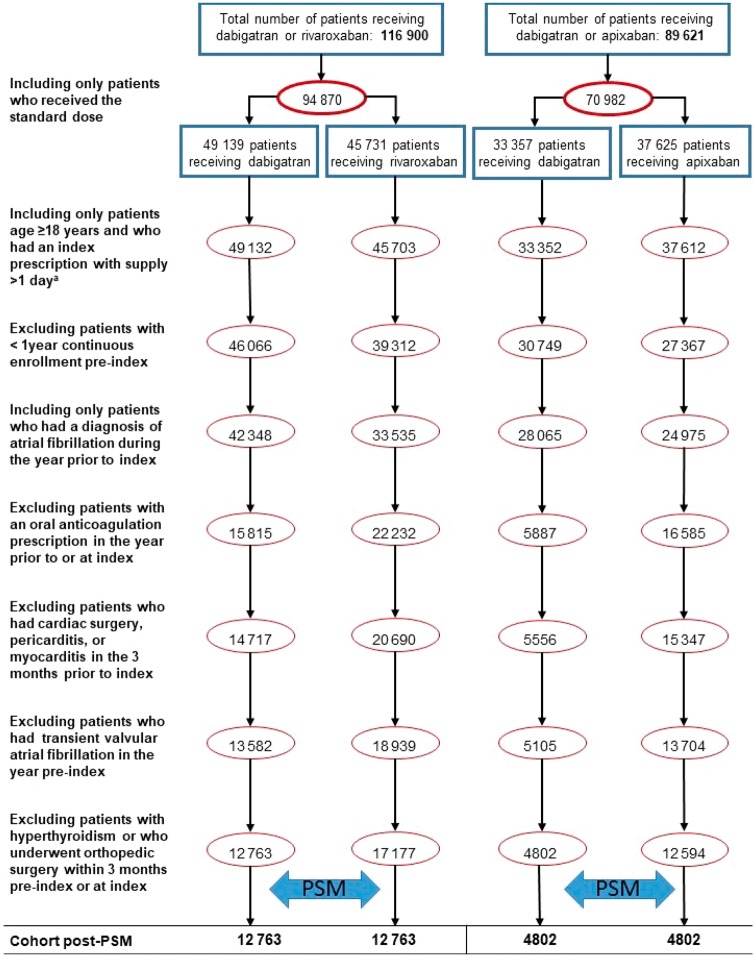
Patient selection and attrition. Dabigatran vs. rivaroxaban (1 July 2011–30 June 2016); dabigatran vs. apixaban (28 December 2012–30 June 2016). ^a^Patients needed ≥2 days of exposure to the index DOAC to ensure they had ≥1 day of index DOAC exposure in the post-index follow-up period. DOAC, direct oral anticoagulant; PSM, propensity score matched.

For the dabigatran vs. rivaroxaban cohort, 116 900 patients had ≥1 prescription for dabigatran or rivaroxaban during the study period, with 94 870 receiving the standard dose (*Figure 1*). After applying inclusion/exclusion criteria, 12 763 patients on dabigatran and 17 177 patients on rivaroxaban were identified (*Figure 1*). Following 1:1 propensity score matching, the final dabigatran vs. rivaroxaban cohort included 12 763 patients on dabigatran and 12 763 patients on rivaroxaban. In this cohort, the mean on-treatment follow-up time for the dabigatran group was 422.1 days (range 2–1827 days), and for the rivaroxaban group it was 417.0 days (range 2–1664 days). Reasons for discontinuation before the study end date are shown in Supplementary data online, *Table S6*.

Prior to propensity score matching, the dabigatran and rivaroxaban groups had similar baseline characteristics (Supplementary data online, *Table S4*). Dabigatran-treated patients were slightly younger (mean age 70.9 ± 10.0 years for dabigatran vs. 71.3 ± 9.7 years for rivaroxaban) and 62% of the dabigatran group were men compared with 61% of the rivaroxaban group.

After propensity score matching, balance between the groups was further improved (*Table 1*). The mean age was 70.9 ± 10.0/10.1 years in the dabigatran and rivaroxaban groups and the proportion of males was 61.9% and 61.4% for the two groups, respectively. Risk factor scores were also well balanced between the groups, with a mean value for CHADS_2_ score of 1.77 for both groups, CHA_2_DS_2_-VASc score of 3.10 for both groups, modified HAS-BLED score of 2.3 for both groups, and Charlson comorbidity index (CCI) of 4.3 for both groups (*Table 1*).

**Figure 2:**
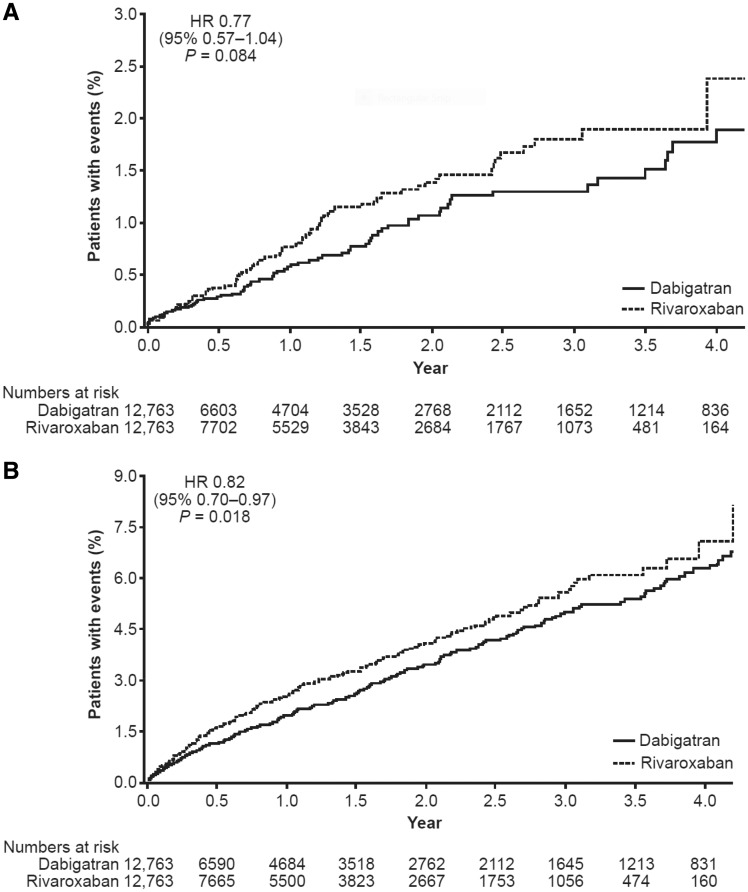
Time from index DOAC to first event (on-treatment analysis): PSM dabigatran vs. rivaroxaban cohort. (*A*) First stroke. (*B*) First major bleeding event. Kaplan–Meier curves are shown. Hazard ratios are based on Cox regression analyses. CI, confidence interval; DOAC, direct oral anticoagulant; HR, hazard ratio; PSM, propensity score matched.

During follow-up, 77 of 12 763 dabigatran patients (0.60%) and 100 of 12 763 rivaroxaban patients (0.78%) had a stroke, giving incidence rates per 100 person-years (95% CI) of 0.52 (0.41–0.66) with dabigatran and 0.69 (0.56–0.84) with rivaroxaban (*Table 2*). As shown in *Table 2* and *Figure 2A*, no significant difference in the risk of stroke was observed between the PSM patients receiving dabigatran or rivaroxaban [hazard ratio (HR) 0.77, 95% CI 0.57–1.04; *P *=* *0.084]. For major bleeding events (*Table 2* and *Figure 2B*), dabigatran was associated with a lower risk compared with rivaroxaban (HR 0.82, 95% CI 0.70–0.97; *P *=* *0.018).

**Figure 3:**
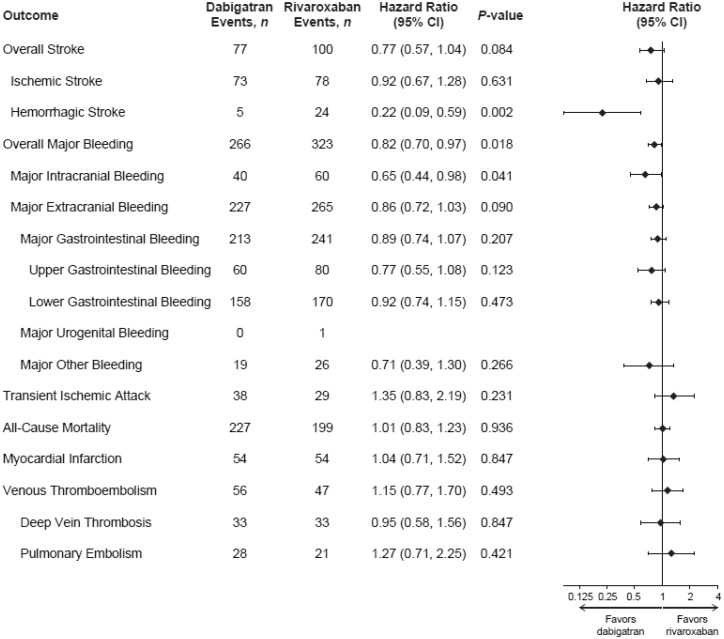
Hazard ratios for first outcome event (on-treatment analysis): PSM dabigatran vs. rivaroxaban cohort. *Note:* numbers of events across subtypes of an outcome may exceed the total number of events for that outcome as a patient can be diagnosed with >1 condition. Cox regression analyses. Hazard ratios were not calculated for major urogenital bleeding. CI, confidence interval; PSM, propensity score matched.

*Figure 3* shows the secondary and additional outcomes in the dabigatran vs. rivaroxaban cohort. Among reported strokes, patients on dabigatran had a similar event rate of ischaemic stroke (HR 0.92, 95% CI 0.67–1.28; *P *=* *0.631), but a lower risk of haemorrhagic stroke (HR 0.22, 95% CI 0.09–0.59; *P *=* *0.002) than patients on rivaroxaban. For the individual component sites of major bleeding events, dabigatran treatment was associated with a lower risk of major intracranial bleeding vs. rivaroxaban (HR 0.65, 95% CI 0.44–0.98; *P *=* *0.041). However, no statistically significant differences were observed in major extracranial bleeding (HR 0.86, 95% CI 0.72–1.03; *P *=* *0.090), nor in all-cause mortality (HR 1.01, 95% CI 0.83–1.23; *P *=* *0.936) between the dabigatran vs. rivaroxaban groups.

### Dabigatran vs. apixaban

In the dabigatran vs. apixaban cohort, 89 621 patients had ≥1 prescription for dabigatran or apixaban during the study period (between 28 December 2012 and 30 June 2016), with 70 982 receiving the standard dose. After applying inclusion/exclusion criteria, 4802 patients taking dabigatran and 12 594 taking apixaban were identified (*Figure 1*). Following 1:1 propensity score matching, the final cohort included 4802 patients on dabigatran and 4802 on apixaban. In this cohort, mean follow-up for the dabigatran-treated patients was 349.5 days (range 2–1280 days) vs. 357.7 days (range 2–1234 days) for the apixaban-treated patients.

Prior to propensity score matching, dabigatran-treated patients were younger (mean age 70.2 ± 10.2 years vs. 72.4 ± 10.0 years for apixaban-treated patients) (Supplementary data online, *Table S5*). Dabigatran-treated patients had lower mean CCI, CHADS_2_, CHA_2_DS_2_-VASc, and modified HAS-BLED scores.

After propensity score matching, patients in the dabigatran and apixaban groups had very similar baseline characteristics (*Table 1*). The mean age for each group was 70.2 years, with 76% being ≥65 years of age. The two groups also had essentially similar mean scores for CHADS_2_ (1.70 for dabigatran vs. 1.69 for apixaban), CHA_2_DS_2_-VASc (2.98 for dabigatran vs. 2.97 for apixaban), modified HAS-BLED (2.3 for both groups), and CCI (4.2 for both groups).

**Figure 4:**
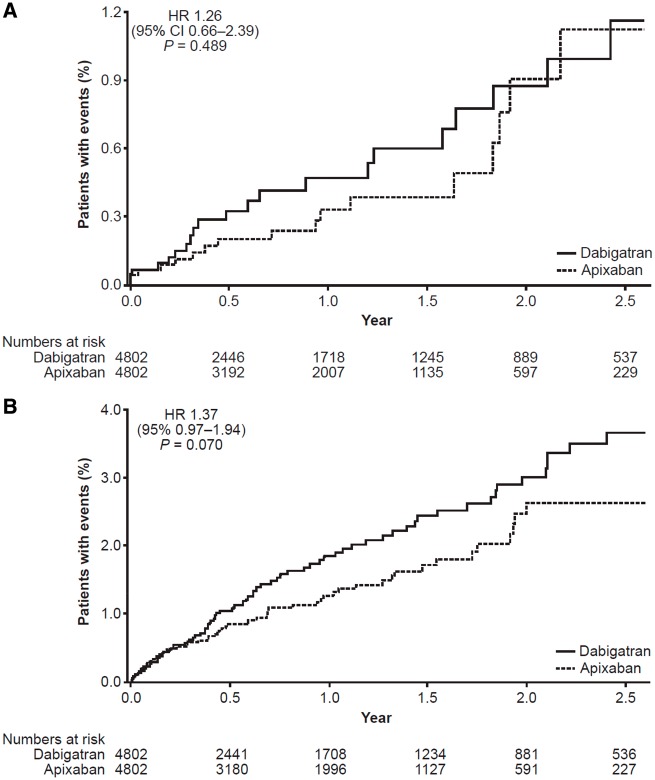
Time from index DOAC to first event on treatment: PSM dabigatran vs. apixaban cohort. (*A*) First stroke. (*B*) First major bleeding event. Kaplan–Meier curves are shown. HRs are based on Cox regression analyses. CI, confidence interval; DOAC, direct oral anticoagulant; HR, hazard ratio; PSM, propensity score matched.

**Figure 5:**
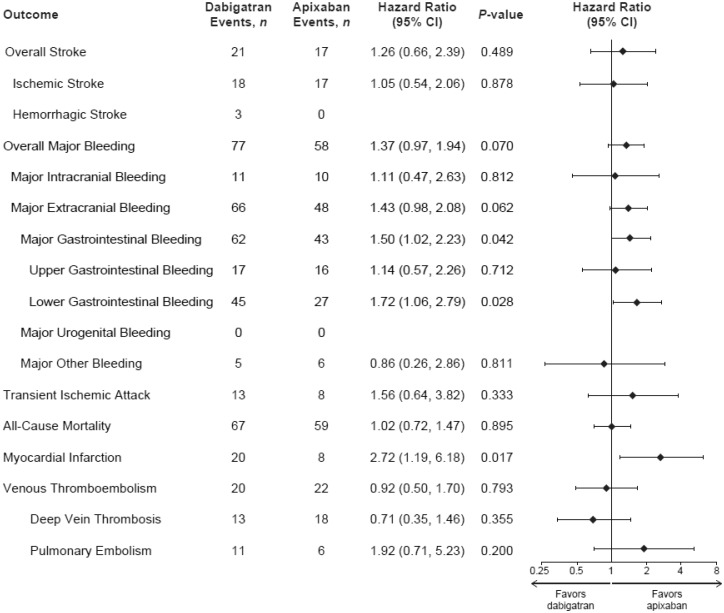
Hazard ratios for first outcome event (on-treatment analysis): PSM dabigatran vs. apixaban cohort. *Note:* numbers of events across subtypes of an outcome may exceed the total number of events for that outcome as one patient can be diagnosed with >1 condition. Cox regression analyses. Hazard ratios were not calculated for haemorrhagic stroke or major urogenital bleeding. CI, confidence interval; PSM, propensity score matched.

The dabigatran vs. apixaban cohort was underpowered, as the minimum sufficient sample size to detect a difference, were a difference to exist, was estimated to be 11 682 dabigatran patients. Therefore, the results that follow should be interpreted with caution. During follow-up, 21 of 4802 dabigatran patients (0.44%) and 17 of 4802 apixaban patients (0.35%) had a stroke, giving incidence rates per 100 person-years (95% CI) of 0.46 (0.28–0.70) with dabigatran and 0.36 (0.21–0.58) with apixaban (*Table 2*). In the dabigatran vs. apixaban PSM cohort, the stroke HR was 1.26 (95% CI 0.66–2.39; *P *=* *0.489; *Table 2* and *Figure 4*). For major bleeding, the HR for dabigatran and apixaban users was 1.37 (95% CI 0.97–1.94; *P *=* *0.070; *Table 2* and *Figure 5*).

In this cohort, the two groups appeared to have similar risk of ischaemic stroke, (HR 1.05, 95% CI 0.54–2.06; *P *=* *0.878) (*Figure 5*). For haemorrhagic stroke, the number of events was low (*n* = 3) and the HR was not estimated. With regard to the individual component sites of major bleeding events, overall no difference was found in the dabigatran vs. apixaban cohort for intracranial bleeding (HR 1.11, 95% CI 0.47–2.63; *P *=* *0.812). There was also no difference in the rates of major extracranial bleeding (HR 1.43, 95% CI 0.98–2.08; *P *=* *0.062). However, there were fewer major GI bleeding events in the apixaban group (HR 1.50, 95% CI 1.02–2.23; *P *=* *0.042). For MI, the HR was 2.72 (95% CI 1.19–6.18; *P *=* *0.017). Looking at all-cause mortality, there were no differences in risk identified between the two groups (HR 1.02, 95% CI 0.72–1.47; *P *=* *0.895).

## Discussion

This observational analysis of the DoD Military Health System clinical database explored the comparative safety and effectiveness of dabigatran vs. rivaroxaban or apixaban in patients with NVAF treated in routine clinical practice. In the dabigatran vs. rivaroxaban cohort, after propensity score matching, there was no significant difference in the risk of stroke for either group. However, we observed a significantly lower risk of major bleeding with dabigatran compared with rivaroxaban; specifically, dabigatran was associated with a significantly lower incidence of intracranial haemorrhage. No differences between groups were identified for all-cause mortality.

In the dabigatran vs. apixaban cohort, the number of patients identified was much lower and, based on our power calculations, the results of the statistical tests for this cohort should be interpreted with caution, and no definitive conclusions can be drawn.

The results from this study are consistent with those of several other recent studies that compared dabigatran with rivaroxaban cohorts.^10^^,^^14^ As mentioned above, a retrospective analysis of PSM patients with NVAF who were ≥65 years of age and newly initiated on either dabigatran or rivaroxaban, found no significant difference in thromboembolic stroke (HR 0.81, 95% CI 0.65–1.01; *P *=* *0.07), and a significant increase in intracranial haemorrhage (HR 1.65, 95% CI 1.20–2.26; *P *=* *0.002) and major extracranial bleeding (HR 1.48, 95% CI 1.32–1.67; *P *<* *0.001) with rivaroxaban compared with dabigatran.^10^ It is important to keep power in mind when interpreting results, as a small sample size not only results in the inability to distinguish between clinically important and unimportant outcomes (wide CIs) and reduces the likelihood to identify moderate differences, but also results in an increased risk of chance results. The probability that a significant result is true depends on the statistical power of the study.^15^ Considering that both low sample size and multiple testing increase the potential for false-positive results, and taking into account the wide CIs, the meaningfulness and robustness of the significant differences observed do not allow for definitive conclusions in the dabigatran vs. apixaban cohort.

The risk for a major bleeding event (requiring hospitalization) among patients with NVAF who were newly initiated on either warfarin, apixaban, dabigatran, or rivaroxaban in clinical practice settings was assessed in an analysis of the Truven MarketScan^®^ Commercial and Medicare supplemental claims database.^14^ Adult patients with NVAF, newly initiating oral anticoagulation after ≥1-year baseline period were identified, and propensity score matching was used to balance patient variables including age, sex, region, baseline comorbidities, and concomitant medications. Of 45 361 newly anticoagulated patients, 34.1% (*n* = 15 461) initiated warfarin, 16.4% (*n* = 7438) initiated apixaban, 39.2% (*n* = 17 801) initiated rivaroxaban, and 10.3% (*n* = 4661) initiated dabigatran.^14^

Among previously published studies, this analysis has the largest number of apixaban patients. However, as with our analysis, limited sample size—and therefore power—also warrants caution in the interpretation of the comparisons to dabigatran. When compared with PSM patients initiating warfarin, significantly lower risks for major bleeding events were reported for those initiating apixaban (*n* = 6964 per group, HR 0.53, 95% CI 0.39–0.71) or dabigatran (*n* = 4515, HR 0.69, 95% CI 0.50–0.96). Patients initiating rivaroxaban demonstrated no significant difference in their risk of major bleeding compared with PSM warfarin patients (*n* = 12 625, HR 0.98, 95% CI 0.83–1.17). Between standard-dose DOACs, PSM patients on rivaroxaban had a significantly higher risk of major bleeding vs. patients on apixaban (*n* = 7399, HR 1.77, 95% CI 1.29–2.45) or dabigatran (*n* = 4657, HR 1.65, 95% CI 1.15–2.36). The risk of major bleeding was similar for dabigatran vs. apixaban (*n* = 4407, HR 0.99, 95% CI 0.64–1.53).^14^ Another recent study looking at a 5% random sample of Medicare claims between 2013 and 2014 had even more limited sample sizes (1415, 2358, and 5139 dabigatran, apixaban, and rivaroxaban users, respectively).^16^

Overall, the effectiveness and safety findings for the novel DOACs in our large patient population are similar to the results reported in retrospective cohort comparisons performed in other real-world populations. Comparisons with apixaban are hampered by the limited sample sizes available. In general, the findings common to these studies are that the rates of stroke and systemic embolism were not significantly different between patients on dabigatran, rivaroxaban, or apixaban, albeit as yet there is no truly adequately powered analysis with apixaban. However, some differences were noted in the risk of major bleeding, with patients treated with dabigatran and apixaban being generally at lower risk than patients on rivaroxaban. A recent meta-analysis of observational studies by Li *et al*.^17^ also found that both apixaban and dabigatran were associated with a similar risk of stroke to rivaroxaban, but with a lower risk of major bleeding events. The analysis also found that the risk of stroke was similar for both dabigatran and apixaban, but that dabigatran was associated with a higher risk of major bleeding. However, the authors noted significant heterogeneity in the risk of major bleeding between studies, with only one of the six individual studies included in the analysis concluding a significant difference in major bleeding events between the two treatments, and this study had included both treatment-naïve and treatment-experienced patients across diverging periods of drug availability.^18^

As with all real-world analyses, our analysis has particular strengths in terms of translation to clinical practice. Due to the wide representation of patients in the DoD database, in terms of both demography and geography, the results of this study are expected to have broad external validity to the US population.^11^^,^^12^ Careful calculation of propensity scores for patients on baseline characteristics, comorbidities, and concomitant medications identified well-matched cohorts. Our study also provided a long on-treatment follow-up duration (mean 422 days for dabigatran vs. rivaroxaban and 350 days for dabigatran vs. apixaban), which was probably facilitated at least in part to the unique stability of enrolment of active duty personnel, retirees, and their families.

Similar to other retrospective database analyses, this study is subject to several inherent limitations including the possibility of coding errors of omission and commission, a lack of central adjudication for events, with use of ICD-9 and ICD-10 codes and claims to identify baseline medical conditions and medications, and incomplete claims.^19^^,^^20^ Despite the standardization of the ICD codes system, there is the potential for incomplete or inaccurate event accounting related to the use of ICD codes to identify events. ICD coding for stroke has been reported to be equally good when using ICD-9 codes (90% positive predictive value, 95% CI 86–93), and ICD-10 codes (92% positive predictive value, 95% CI 88–95).^21^ Similarly, an analysis of algorithms based on ICD-9 codes, which were used to define treatment outcomes (including intracranial haemorrhage, major extracranial bleeding, and major GI bleeding), reported positive predictive values ranging from 86% to 97%.^10^

Furthermore, as with all non-randomized studies, imbalance in unmeasured prognostic factors could bias the results (residual confounding). Our study design minimized this risk by restricting inclusion to treatment-naïve patients initiating treatment in the period of common treatment availability following the approval date of both compared medications, and the use of propensity score methods, a very powerful method for controlling for confounding if proper variables and data are utilized in the analysis.^22^

## Conclusions

Overall, this analysis showed that patients in the DoD Military Health System Data Repository who were newly initiated on dabigatran had a statistically significantly lower risk of major bleeding than patients newly starting rivaroxaban, while the risk of stroke was not significantly different. For the cohort comparing dabigatran vs. apixaban, the reduced sample size limits the ability to draw definitive conclusions. In the absence of head-to-head clinical trials comparing available DOACs, this practice-based analysis of direct comparisons of patient outcomes data may help inform clinical decision-making.

## Supplementary Material

Supplementary Information
