# Supplementary material for: Comparative safety and effectiveness of dabigatran vs. rivaroxaban and apixaban in patients with non-valvular atrial fibrillation: a retrospective study from a large healthcare system
Source: Eur Heart J Cardiovasc Pharmacother. 2018 Nov 30;5(2):80–90. doi: 10.1093/ehjcvp/pvy044 (PMC6418470; doi:10.1093/ehjcvp/pvy044)
Supplement: Supplementary Information [file pvy044_supplementary_information.pdf]

Supplementary Information

Table 1S. Exclusion diagnoses: list of codes and descriptions.

**Exclusion diagnosis**	**ICD-9 diagnosis codes**	**ICD-10 diagnosis codes**
Hyperthyroidism	242.x	E05.00, E05.01, E05.10, E05.11, E05.20, E05.21, E05.30, E05.31, E05.40, E05.41, E05.80, E05.81, E05.90, E05.91
Orthopedic procedures (See also procedure codes, in supplementary Table 2)	V43.64, V43.65	Z9664x, Z9665x
Deep vein thrombosis	451.xx, 453.xx	I80.xxx, I82.xxx
Pulmonary embolism	415.1x	I26.01, I26.09, I26.90, I26.92, I26.99, I27.82
Pericarditis	391.x, 393, 420.x, 423.2, 036.41, 074.21, 093.81, 098.83	A39.53, B33.23, I01.0, I09.2, I30.0, I30.1, I30.8, I30.9, I31.0, I32, M32.12
Myocarditis	391.2, 422.xx, 074.23, 398.0, 429.0, 032.82, 036.43, 093.82, 130.3	A36.81, A38.1, A39.52, A52.06, A54.83, B26.82, B33.22, B58.81, D86.85, I01.2, I09.0, I40.0, I40.1, I40.8, I41, I511.4, J10.82, J11.82
Valvular atrial fibrillation		
Mitral stenosis	394.0x	I05.0, I05.1, I05.2, I05.8, I05.9, I34.2
Mitral stenosis with insufficiency	394.2	I34.0, I34.2, I34.8, I34.9
Mitral valve stenosis and aortic valve stenosis	396.0	I06.0, I35.0, Q23.0
Mitral valve stenosis and aortic valve insufficiency	396.1	I08.0, I06.1, I25.1, Q23.1, Q23.8, Q23.9
Diseases of other endocardial structures	397.x	I07.x, I09.89, I08.1, I08.2, I08.3, I08.8, I08.9
Other and unspecified rheumatic heart diseases	398.9x	I09.9, I09.81, I09.89

Heart valve replaced by transplant	V42.2	Z95.3, Z95.4
Heart valve replaced by a mechanical device/prosthesis	V43.3	Z95.2
Atrioventricular valve repair	V43.3	Z95.2
Aortic valve valvuloplasty/diseases of aortic valve	395.x	I06.x

Table 2S. Exclusion procedures: codes and descriptions.

	Procedure codes		
**Exclusion procedure**	**ICD-9**	**ICD-10**	**CPT**
Valvular atrial fibrillation procedure/CPT codes			
Open heart valvuloplasty without replacement	35.1x	02QF0ZZ, 02QG0ZZ, 02QH0ZZ, 02QJ0ZZ, 027F04Z, 027F0DZ, 027F0ZZ, 02NF0ZZ, 02QF0ZZ, 027G04Z, 027G0DZ, 027G0ZZ, 02NG0ZZ, 02QG0ZZ, 027H04Z, 027H0DZ, 027H0ZZ, 02NH0ZZ, 02QH0ZZ, 027J04Z, 027J0DZ, 027J0ZZ, 02NJ0ZZ, 02QJ0ZZ	
Open and other replacement of heart valve (includes: open and other replacement of unspecified heart valve, open and other replacement of aortic valve, open and other replacement of mitral valve, open and other replacement of mitral valve, open and other replacement of pulmonary valve, open and other replacement of tricuspid valve.)	35.2x	02RF07Z, 02RF08Z, 02RF0JZ, 02RF0KZ, 02RF47Z, 02RF48Z, 02RF4JZ, 02RF4KZ, 02RG07Z, 02RG08Z, 02RG0JZ, 02RG0KZ, 02RG47Z, 02RG48Z, 02RG4JZ, 02RG4KZ, 02RH07Z, 02RH08Z, 02RH0JZ, 02RH0KZ, 02RH47Z, 02RH48Z, 02RH4JZ, 02RH4KZ, 02RJ07Z, 02RJ08Z, 02RJ0JZ, 02RJ0KZ, 02RJ47Z, 02RJ48Z, 02RJ4JZ, 02RJ4KZ, 02RF07Z, 02RF08Z, 02RF0KZ, 02RF47Z, 02RF48Z, 02RF4KZ, X2RF032, X2RF432, 02RF0JZ, 02RF4JZ, 02RG07Z, 02RG08Z, 02RG0KZ, 02RG37Z, 02RG38Z, 02RG3KZ, 02RG47Z, 02RG48Z, 02RG4KZ, 02RG0JZ, 02RG3JZ, 02RG4JZ, 02RH07Z, 02RH08Z, 02RH0KZ, 02RH47Z, 02RH48Z, 02RH4KZ, 02RH0JZ, 02RH4JZ, 02RJ07Z, 02RJ08Z, 02RJ0KZ, 02RJ47Z, 02RJ48Z, 02RJ4KZ, 02RJ0JZ, 02RJ4JZ	
Closed heart valvotomy	35.0x	02NF3ZZ, 02NF4ZZ, 02NG3ZZ, 02NG4ZZ, 02NH3ZZ, 02NH4ZZ, 02NJ3ZZ, 02NJ4ZZ, 02NF3ZZ, 02NF4ZZ, 02NG3ZZ, 02NG4ZZ, 02NH3ZZ, 02NH4ZZ, 02NJ3ZZ, 02NJ4ZZ, 02RF37Z, 02RF38Z, 02RF3JZ, 02RF3KZ, X2RF332, 02RF37H, 02RF38H, 02RF3JH, 02RF3KH, 02RH37Z, 02RH38Z, 02RH3JZ, 02RH3KZ, 02RH37H, 02RH38H, 02RH3JH, 02RH3KH, 02RF37Z, 02RF38Z, 02RF3JZ, 02RF3KZ, 02RG37H, 02RG37Z, 02RG38H,	

		02RG38Z, 02RG3JH, 02RG3JZ, 02RG3KH, 02RG3KZ, 02RH37Z, 02RH38Z, 02RH3JZ, 02RH3KZ	
Unlisted procedure, cardiac surgery			33999
Implantation of catheter-delivered prosthetic aortic heart valve; open thoracic approach			0257T
Transthoracic cardiac exposure (e.g., sternotomy, thoracotomy, subxiphoid) for catheter-delivered aortic valve replacement; without cardiopulmonary bypass			0258T
Transthoracic cardiac exposure (e.g., sternotomy, thoracotomy, subxiphoid) for catheter-delivered aortic valve replacement; with cardiopulmonary bypass			0259T
Replacement, aortic valve, with cardiopulmonary bypass; with prosthetic valve other than homograft or stentless valve			33405
Valvuloplasty, mitral valve, with cardiopulmonary bypass			33425
Valvuloplasty, mitral valve, with cardiopulmonary bypass; with prosthetic ring			33426

Valvuloplasty, mitral valve, with cardiopulmonary bypass; radical reconstruction, with or without ring			33427
Replacement, mitral valve, with cardiopulmonary bypass			33430
Implantation of catheter-delivered prosthetic pulmonary valve, endovascular approach			0262T
Replacement, pulmonary valve			33475
Valvectomy, tricuspid valve, with cardiopulmonary bypass			33460
Valvuloplasty, tricuspid valve; without ring insertion			33463
Valvuloplasty, tricuspid valve; with ring insertion			33464
Replacement, tricuspid valve, with cardiopulmonary bypass			33465
Description for CPT=33660-33665			33660-33665
Description for CPT=33400-33403			33400-33403
Orthopedic procedures (hip/knee)			

Total/partial hip replacement	81.52	0SRA009, 0SRA00A, 0SRA00Z, 0SRA019, 0SRA01A, 0SRA01Z, 0SRA039, 0SRA03A, 0SRA03Z, 0SRA07Z, 0SRA0J9, 0SRA0JA, 0SRA0JZ, 0SRA0KZ, 0SRE009, 0SRE00A, 0SRE00Z, 0SRE019, 0SRE01A, 0SRE01Z, 0SRE039, 0SRE03A, 0SRE03Z, 0SRE07Z, 0SRE0J9, 0SRE0JA, 0SRE0JZ, 0SRE0KZ, 0SRR019, 0SRR01A, 0SRR01Z, 0SRR039, 0SRR03A, 0SRR03Z, 0SRR07Z, 0SRR0J9, 0SRR0JA, 0SRR0JZ, 0SRR0KZ, 0SRS019, 0SRS01A, 0SRS01Z, 0SRS039, 0SRS03A, 0SRS03Z, 0SRS07Z, 0SRS0J9, 0SRS0JA, 0SRS0JZ	
	81.51	0SR90J9, 0SR90JA, 0SR90JZ, 0SRB0J9, 0SRB0JA, 0SRB0JZ	
	0070, 0071, 0072, 0073, 8153	0SP908Z, 0SP909Z, 0SP90BZ, 0SP90JZ, 0SPB08Z, 0SPB09Z, 0SPB0BZ, 0SPB0JZ, 0SR9019, 0SR901A, 0SR901Z, 0SR9029, 0SR902A, 0SR902Z, 0SR9039, 0SR903A, 0SR903Z, 0SR9049, 0SR904A, 0SR904Z, 0SR90J9, 0SR90JA, 0SR90JZ, 0SRB019, 0SRB01A, 0SRB01Z, 0SRB029, 0SRB02A, 0SRB02Z, 0SRB039, 0SRB03A, 0SRB03Z, 0SRB049, 0SRB04A, 0SRB04Z, 0SRB0J9, 0SRB0JA, 0SRB0JZ, 0SP908Z, 0SP909Z, 0SP90BZ, 0SPA0JZ, 0SPB08Z, 0SPB09Z, 0SPB0BZ, 0SPE0JZ, 0SRA009, 0SRA00A, 0SRA00Z, 0SRA019, 0SRA01A, 0SRA01Z, 0SRA039, 0SRA03A, 0SRA03Z, 0SRA0J9, 0SRA0JA, 0SRA0JZ, 0SRE009, 0SRE00A, 0SRE00Z, 0SRE019, 0SRE01A, 0SRE01Z, 0SRE039, 0SRE03A, 0SRE03Z, 0SRE0J9, 0SRE0JA, 0SRE0JZ, 0SP908Z, 0SP909Z, 0SP90BZ, 0SPB08Z, 0SPB09Z, 0SPB0BZ, 0SPR0JZ, 0SPS0JZ, 0SRR019, 0SRR01A, 0SRR01Z, 0SRR039, 0SRR03A, 0SRR03Z, 0SRR0J9, 0SRR0JA, 0SRR0JZ, 0SRS019, 0SRS01A, 0SRS01Z, 0SRS039, 0SRS03A, 0SRS03Z, 0SRS0J9, 0SRS0JA, 0SRS0JZ, 0SP908Z, 0SP909Z, 0SP90BZ, 0SP90JZ, 0SPB08Z, 0SPB09Z, 0SPB0BZ, 0SPB0JZ, 0SU909Z, 0SUA09Z, 0SUB09Z, 0SUE09Z, 0SUR09Z, 0SUS09Z, 0SW90JZ, 0SW93JZ, 0SW94JZ, 0SWA0JZ, 0SWA3JZ, 0SWA4JZ, 0SWB0JZ, 0SWB3JZ, 0SWB4JZ, 0SWE0JZ, 0SWE3JZ, 0SWE4JZ, 0SWR0JZ, 0SWR3JZ, 0SWR4JZ, 0SWS0JZ, 0SWS3JZ, 0SWS4JZ	

			27125, 27130, 27132, 27134, 27137, 27138
Total/partial knee replacement	81.54	0SRC07Z, 0SRC0JZ, 0SRC0KZ, 0SRC0LZ, 0SRD07Z, 0SRD0JZ, 0SRD0KZ, 0SRD0LZ, 0SRT07Z, 0SRT0JZ, 0SRT0KZ, 0SRU07Z, 0SRU0JZ, 0SRU0KZ, 0SRV07Z, 0SRV0JZ, 0SRV0KZ, 0SRW07Z, 0SRW0JZ, 0SRW0KZ	
	0080, 0081, 0082, 0083, 0084, 8155	0SPC08Z, 0SPC09Z, 0SPC0JZ, 0SPC48Z, 0SPC4JZ, 0SPD08Z, 0SPD09Z, 0SPD0JZ, 0SPD48Z, 0SPD4JZ, 0SRC0J9, 0SRC0JA, 0SRC0JZ, 0SRD0J9, 0SRD0JA, 0SRD0JZ, 0SPC08Z, 0SPC09Z, 0SPC48Z, 0SPC4JZ, 0SPD08Z, 0SPD09Z, 0SPD48Z, 0SPD4JZ, 0SPT0JZ, 0SPU0JZ, 0SRV0J9, 0SRV0JA, 0SRV0JZ, 0SRW0J9, 0SRW0JA, 0SRW0JZ, 0SPC08Z, 0SPC09Z, 0SPC48Z, 0SPC4JZ, 0SPD08Z, 0SPD09Z, 0SPD48Z, 0SPD4JZ, 0SPV0JZ, 0SPW0JZ, 0SRT0J9, 0SRT0JA, 0SRT0JZ, 0SRU0J9, 0SRU0JA, 0SRU0JZ, 0QPD0JZ, 0QPD3JZ, 0QPD4JZ, 0QPF0JZ, 0QPF3JZ, 0QPF4JZ, 0QRD0JZ, 0QRD3JZ, 0QRD4JZ, 0QRF0JZ, 0QRF3JZ, 0QRF4JZ, 0QUD0JZ, 0QUD3JZ, 0QUD4JZ, 0QUF0JZ, 0QUF3JZ, 0QUF4JZ, 0SUC09C, 0SUD09C, 0SPC09Z, 0SPD09Z, 0SUV09Z, 0SUW09Z, 0SWC0JC, 0SWC0JZ, 0SWC3JC, 0SWC3JZ, 0SWC4JC, 0SWC4JZ, 0SWD0JC, 0SWD0JZ, 0SWD3JC, 0SWD3JZ, 0SWD4JC, 0SWD4JZ, 0SWT0JZ, 0SWT3JZ, 0SWT4JZ, 0SWU0JZ, 0SWU3JZ, 0SWU4JZ, 0SWV0JZ, 0SWV3JZ, 0SWV4JZ, 0SWW0JZ, 0SWW3JZ, 0SWW4JZ	
			27447, 27486, 27487, 27437, 27438, 27440, 27441, 27442, 27443, 27445, 27446

Cardiac surgery	00.5x, 35.xx, 36.xx, 37.xx	0210083, 0210088, 0210089, 021008C, 021008F, 021008W, 0210093, 0210098, 0210099, 021009C, 021009F, 021009W, 02100A3, 02100A8, 02100A9, 02100AC, 02100AF, 02100AW, 02100J3, 02100J8, 02100J9, 02100JC, 02100JF, 02100JW, 02100K3, 02100K8, 02100K9, 02100KC, 02100KF, 02100KW, 02100Z3, 02100Z8, 02100Z9, 02100ZC, 02100ZF, 0210344, 02103D4, 0210444, 0210483, 0210488, 0210489, 021048C, 021048F, 021048W, 0210493, 0210498, 0210499, 021049C, 021049F, 021049W,	
		02104A3, 02104A8, 02104A9, 02104AC, 02104AF, 02104AW, 02104D4, 02104J3, 02104J8, 02104J9, 02104JC, 02104JF, 02104JW, 02104K3, 02104K8, 02104K9, 02104KC, 02104KF, 02104KW, 02104Z3, 02104Z8, 02104Z9, 02104ZC, 02104ZF, 0211083, 0211088, 0211089, 021108C, 021108F, 021108W, 0211093, 0211098, 0211099, 021109C, 021109F, 021109W, 02110A3, 02110A8, 02110A9, 02110AC, 02110AF, 02110AW, 02110J3, 02110J8, 02110J9, 02110JC, 02110JF, 02110JW, 02110K3, 02110K8, 02110K9, 02110KC, 02110KF, 02110KW, 02110Z3, 02110Z8, 02110Z9, 02110ZC, 02110ZF, 0211344,	
		02113D4, 0211444, 0211483, 0211488, 0211489, 021148C, 021148F, 021148W, 0211493, 0211498, 0211499, 021149C, 021149F, 021149W, 02114A3, 02114A8, 02114A9, 02114AC, 02114AF, 02114AW, 02114D4, 02114J3, 02114J8, 02114J9, 02114JC, 02114JF, 02114JW, 02114K3, 02114K8, 02114K9, 02114KC, 02114KF, 02114KW, 02114Z3, 02114Z8, 02114Z9, 02114ZC, 02114ZF, 0212083, 0212088, 0212089, 021208C, 021208F, 021208W, 0212093, 0212098, 0212099, 021209C, 021209F, 021209W, 02120A3, 02120A8, 02120A9, 02120AC, 02120AF, 02120AW, 02120J3, 02120J8, 02120J9, 02120JC,	

		02120JF, 02120JW, 02120K3, 02120K8, 02120K9, 02120KC, 02120KF, 02120KW, 02120Z3, 02120Z8, 02120Z9, 02120ZC, 02120ZF, 0212344, 02123D4, 0212444, 0212483, 0212488, 0212489, 021248C, 021248F, 021248W, 0212493, 0212498, 0212499, 021249C, 021249F, 021249W, 02124A3, 02124A8, 02124A9, 02124AC, 02124AF, 02124AW, 02124D4, 02124J3, 02124J8, 02124J9, 02124JC, 02124JF, 02124JW, 02124K3, 02124K8, 02124K9, 02124KC, 02124KF, 02124KW, 02124Z3, 02124Z8, 02124Z9, 02124ZC, 02124ZF, 0213083, 0213088, 0213089, 021308C, 021308F, 021308W, 0213093, 0213098, 0213099, 021309C, 021309F, 021309W, 02130A3, 02130A8, 02130A9, 02130AC, 02130AF, 02130AW,	
		02130J3, 02130J8, 02130J9, 02130JC, 02130JF, 02130JW, 02130K3, 02130K8, 02130K9, 02130KC, 02130KF, 02130KW, 02130Z3, 02130Z8, 02130Z9, 02130ZC, 02130ZF, 0213344, 02133D4, 0213444, 0213483, 0213488, 0213489, 021348C, 021348F, 021348W, 0213493, 0213498, 0213499, 021349C, 021349F, 021349W, 02134A3, 02134A8, 02134A9, 02134AC, 02134AF, 02134AW, 02134D4, 02134J3, 02134J8, 02134J9, 02134JC, 02134JF, 02134JW, 02134K3, 02134K8, 02134K9, 02134KC, 02134KF, 02134KW, 02134Z3, 02134Z8, 02134Z9, 02134ZC, 02134ZF, 021608P, 021608Q, 021608R, 021609P,	

		021609Q, 021609R, 02160AP, 02160AQ, 02160AR, 02160JP, 02160JQ, 02160JR, 02160KP, 02160KQ, 02160KR, 02160Z7, 02160ZP, 02160ZQ, 02160ZR, 021648P, 021648Q, 021648R, 021649P, 021649Q, 021649R, 02164AP, 02164AQ, 02164AR, 02164JP, 02164JQ, 02164JR, 02164KP, 02164KQ, 02164KR, 02164Z7, 02164ZP, 02164ZQ, 02164ZR, 021708P, 021708Q, 021708R, 021708S, 021708T, 021708U, 021709P, 021709Q, 021709R, 021709S, 021709T, 021709U, 02170AP, 02170AQ, 02170AR, 02170AS, 02170AT, 02170AU, 02170JP, 02170JQ, 02170JR, 02170JS, 02170JT, 02170JU, 02170KP, 02170KQ, 02170KR, 02170KS, 02170KT, 02170KU, 02170ZP, 02170ZQ, 02170ZR, 02170ZS, 02170ZT, 02170ZU,	
		021748P, 021748Q, 021748R, 021748S, 021748T, 021748U, 021749P, 021749Q, 021749R, 021749S, 021749T, 021749U, 02174AP, 02174AQ, 02174AR, 02174AS, 02174AT, 02174AU, 02174JP, 02174JQ, 02174JR, 02174JS, 02174JT, 02174JU, 02174KP, 02174KQ, 02174KR, 02174KS, 02174KT, 02174KU, 02174ZP, 02174ZQ, 02174ZR, 02174ZS, 02174ZT, 02174ZU, 021K08P, 021K08Q, 021K08R, 021K09P, 021K09Q, 021K09R, 021K0AP, 021K0AQ, 021K0AR, 021K0JP, 021K0JQ, 021K0JR, 021K0KP, 021K0KQ, 021K0KR, 021K0Z5, 021K0Z8, 021K0Z9, 021K0ZC, 021K0ZF, 021K0ZP, 021K0ZQ, 021K0ZR, 021K0ZW,	
		021K48P, 021K48Q, 021K48R, 021K49P, 021K49Q, 021K49R, 021K4AP, 021K4AQ, 021K4AR, 021K4JP, 021K4JQ, 021K4JR, 021K4KP, 021K4KQ, 021K4KR, 021K4Z5, 021K4Z8, 021K4Z9, 021K4ZC, 021K4ZF, 021K4ZP, 021K4ZQ, 021K4ZR, 021K4ZW, 021L08P, 021L08Q, 021L08R, 021L09P, 021L09Q, 021L09R, 021L0AP, 021L0AQ, 021L0AR, 021L0JP, 021L0JQ, 021L0JR, 021L0KP, 021L0KQ, 021L0KR, 021L0Z5, 021L0Z8, 021L0Z9, 021L0ZC, 021L0ZF, 021L0ZP, 021L0ZQ, 021L0ZR, 021L0ZW, 021L48P, 021L48Q, 021L48R, 021L49P, 021L49Q, 021L49R, 021L4AP, 021L4AQ, 021L4AR, 021L4JP, 021L4JQ, 021L4JR,	

		021L4KP, 021L4KQ, 021L4KR, 021L4Z5, 021L4Z8, 021L4Z9, 021L4ZC, 021L4ZF, 021L4ZP, 021L4ZQ, 021L4ZR, 021L4ZW, 021V08S, 021V08T, 021V08U, 021V09S, 021V09T, 021V09U, 021V0AS, 021V0AT, 021V0AU, 021V0JS, 021V0JT, 021V0JU, 021V0KS, 021V0KT, 021V0KU, 021V0ZS, 021V0ZT, 021V0ZU, 021V48S, 021V48T, 021V48U, 021V49S, 021V49T, 021V49U, 021V4AS, 021V4AT, 021V4AU, 021V4JS, 021V4JT, 021V4JU, 021V4KS, 021V4KT, 021V4KU, 021V4ZS, 021V4ZT, 021V4ZU, 024F07J, 024F08J, 024F0JJ, 024F0KJ, 024G072, 024G082, 024G0J2, 024G0K2, 024J072, 024J082, 024J0J2, 024J0K2, 02540ZZ, 02543ZZ, 02544ZZ, 02550ZZ, 02553ZZ, 02554ZZ,	
		02560ZZ, 02563ZZ, 02564ZZ, 02570ZK, 02570ZZ, 02573ZK, 02573ZZ, 02574ZK, 02574ZZ, 02580ZZ, 02583ZZ, 02584ZZ, 02590ZZ, 02593ZZ, 02594ZZ, 025D0ZZ, 025D3ZZ, 025D4ZZ, 025F0ZZ, 025F3ZZ, 025F4ZZ, 025G0ZZ, 025G3ZZ, 025G4ZZ, 025H0ZZ, 025H3ZZ, 025H4ZZ, 025J0ZZ, 025J3ZZ, 025J4ZZ, 025K0ZZ, 025K3ZZ, 025K4ZZ, 025L0ZZ, 025L3ZZ, 025L4ZZ, 025M0ZZ, 025M3ZZ, 025M4ZZ, 025N0ZZ, 025N3ZZ, 025N4ZZ, 0270046, 027004Z, 0270056, 027005Z, 0270066, 027006Z, 0270076, 027007Z, 02700D6, 02700DZ, 02700E6, 02700EZ, 02700F6, 02700FZ, 02700G6,	
		02700GZ, 02700T6, 02700TZ, 02700Z6, 02700ZZ, 0270346, 027034Z, 0270356, 027035Z, 0270366, 027036Z, 0270376, 027037Z, 02703D6, 02703DZ, 02703E6, 02703EZ, 02703F6, 02703FZ, 02703G6, 02703GZ, 02703T6, 02703TZ, 0270446, 027044Z, 0270456, 027045Z, 0270466, 027046Z, 0270476, 027047Z, 02704D6, 02704DZ, 02704E6, 02704EZ, 02704F6, 02704FZ, 02704G6, 02704GZ, 02704T6, 02704TZ, 0271046, 027104Z, 0271056, 027105Z, 0271066, 027106Z, 0271076, 027107Z, 02710D6, 02710DZ, 02710E6, 02710EZ, 02710F6, 02710FZ,	

		02710G6, 02710GZ, 02710T6, 02710TZ, 02710Z6, 02710ZZ, 0271346, 027134Z,	
		0271356, 027135Z, 0271366, 027136Z, 0271376, 027137Z, 02713D6, 02713DZ, 02713E6, 02713EZ, 02713F6, 02713FZ, 02713G6, 02713GZ, 02713T6, 02713TZ, 0271446, 027144Z, 0271456, 027145Z, 0271466, 027146Z, 0271476, 027147Z, 02714D6, 02714DZ, 02714E6, 02714EZ, 02714F6, 02714FZ, 02714G6, 02714GZ, 02714T6, 02714TZ, 0272046, 027204Z, 0272056, 027205Z, 0272066, 027206Z, 0272076, 027207Z, 02720D6, 02720DZ, 02720E6, 02720EZ, 02720F6, 02720FZ, 02720G6, 02720GZ, 02720T6, 02720TZ, 02720Z6, 02720ZZ,	
		0272346, 027234Z, 0272356, 027235Z, 0272366, 027236Z, 0272376, 027237Z, 02723D6, 02723DZ, 02723E6, 02723EZ, 02723F6, 02723FZ, 02723G6, 02723GZ, 02723T6, 02723TZ, 0272446, 027244Z, 0272456, 027245Z, 0272466, 027246Z, 0272476, 027247Z, 02724D6, 02724DZ, 02724E6, 02724EZ, 02724F6, 02724FZ, 02724G6, 02724GZ, 02724T6, 02724TZ, 0273046, 027304Z, 0273056, 027305Z, 0273066, 027306Z, 0273076, 027307Z, 02730D6, 02730DZ, 02730E6, 02730EZ, 02730F6, 02730FZ, 02730G6, 02730GZ, 02730T6, 02730TZ, 02730Z6, 02730ZZ, 0273346, 027334Z, 0273356, 027335Z, 0273366, 027336Z, 0273376, 027337Z, 02733D6, 02733DZ, 02733E6, 02733EZ, 02733F6, 02733FZ,	

		02733G6, 02733GZ, 02733T6, 02733TZ, 0273446, 027344Z, 0273456, 027345Z, 0273466, 027346Z, 0273476, 027347Z, 02734D6, 02734DZ, 02734E6, 02734EZ, 02734F6, 02734FZ, 02734G6, 02734GZ, 02734T6, 02734TZ, 027F04Z, 027F0DZ, 027F0ZZ, 027F34Z, 027F3DZ, 027F3ZZ, 027F44Z, 027F4DZ, 027F4ZZ, 027G04Z, 027G0DZ, 027G0ZZ, 027G34Z, 027G3DZ, 027G3ZZ, 027G44Z, 027G4DZ, 027G4ZZ, 027H04Z, 027H0DZ, 027H0ZZ, 027H34Z, 027H3DZ, 027H3ZZ, 027H44Z, 027H4DZ, 027H4ZZ, 027J04Z, 027J0DZ, 027J0ZZ, 027J34Z, 027J3DZ, 027J3ZZ, 027J44Z, 027J4DZ, 027J4ZZ, 027K04Z, 027K0DZ, 027K0ZZ,	
		027K34Z, 027K3DZ, 027K3ZZ, 027K44Z, 027K4DZ, 027K4ZZ, 027P04Z, 027P34Z, 027P44Z, 027Q04Z, 027Q34Z, 027Q44Z, 027R04T, 027R04Z, 027R0DT, 027R0ZT, 027R34T, 027R34Z, 027R3DT, 027R3ZT, 027R44T, 027R44Z, 027R4DT, 027R4ZT, 027S04Z, 027S34Z, 027S44Z, 027T04Z, 027T34Z, 027T44Z, 027V04Z, 027V34Z, 027V44Z, 027W04Z, 027W34Z, 027W44Z, 027X04Z, 027X34Z, 027X44Z, 02880ZZ, 02883ZZ, 02884ZZ, 02890ZZ, 02893ZZ, 02894ZZ, 028D0ZZ, 028D3ZZ, 028D4ZZ, 02B40ZX, 02B40ZZ, 02B43ZX, 02B43ZZ, 02B44ZX, 02B44ZZ, 02B50ZX, 02B50ZZ, 02B53ZX, 02B53ZZ, 02B54ZX, 02B54ZZ,	
		02B60ZX, 02B60ZZ, 02B63ZX, 02B63ZZ, 02B64ZX, 02B64ZZ, 02B70ZK, 02B70ZX, 02B70ZZ, 02B73ZK, 02B73ZX, 02B73ZZ, 02B74ZK, 02B74ZX, 02B74ZZ, 02B80ZX, 02B80ZZ, 02B83ZX, 02B83ZZ, 02B84ZX, 02B84ZZ, 02B90ZX, 02B90ZZ, 02B93ZX, 02B93ZZ, 02B94ZX, 02B94ZZ, 02BD0ZX, 02BD0ZZ, 02BD3ZX, 02BD3ZZ, 02BD4ZX, 02BD4ZZ, 02BF0ZX, 02BF0ZZ, 02BF3ZX, 02BF3ZZ, 02BF4ZX, 02BF4ZZ, 02BG0ZX, 02BG0ZZ, 02BG3ZX, 02BG3ZZ, 02BG4ZX, 02BG4ZZ, 02BH0ZX, 02BH0ZZ, 02BH3ZX, 02BH3ZZ, 02BH4ZX, 02BH4ZZ, 02BJ0ZX, 02BJ0ZZ, 02BJ3ZX, 02BJ3ZZ, 02BJ4ZX, 02BJ4ZZ, 02BK0ZX, 02BK0ZZ, 02BK3ZX,	

		02BK3ZZ, 02BK4ZX, 02BK4ZZ, 02BL0ZX, 02BL0ZZ, 02BL3ZX, 02BL3ZZ, 02BL4ZX, 02BL4ZZ, 02BM0ZX, 02BM0ZZ, 02BM3ZX, 02BM3ZZ, 02BM4ZX, 02BM4ZZ, 02BN0ZX, 02BN0ZZ, 02BN3ZX, 02BN3ZZ, 02BN4ZX, 02BN4ZZ, 02C00Z6, 02C00ZZ, 02C03Z6, 02C03ZZ, 02C04Z6, 02C04ZZ, 02C10Z6, 02C10ZZ, 02C13Z6, 02C13ZZ, 02C14Z6, 02C14ZZ, 02C20Z6, 02C20ZZ, 02C23Z6, 02C23ZZ, 02C24Z6, 02C24ZZ, 02C30Z6, 02C30ZZ, 02C33Z6, 02C33ZZ, 02C34Z6, 02C34ZZ, 02C40ZZ, 02C43ZZ, 02C44ZZ, 02C50ZZ, 02C53ZZ, 02C54ZZ, 02C60ZZ, 02C63ZZ, 02C64ZZ, 02C70ZZ, 02C73ZZ, 02C74ZZ, 02C80ZZ, 02C83ZZ,	
		02C84ZZ, 02C90ZZ, 02C93ZZ, 02C94ZZ, 02CD0ZZ, 02CD3ZZ, 02CD4ZZ, 02CF0ZZ, 02CF3ZZ, 02CF4ZZ, 02CG0ZZ, 02CG3ZZ, 02CG4ZZ, 02CH0ZZ, 02CH3ZZ, 02CH4ZZ, 02CJ0ZZ, 02CJ3ZZ, 02CJ4ZZ, 02CK0ZZ, 02CK3ZZ, 02CK4ZZ, 02CL0ZZ, 02CL3ZZ, 02CL4ZZ, 02CM0ZZ, 02CM3ZZ, 02CM4ZZ, 02CN0ZZ, 02CN3ZZ, 02CN4ZZ, 02FN0ZZ, 02FN3ZZ, 02FN4ZZ, 02H402Z, 02H403Z, 02H40DZ, 02H40JZ, 02H40KZ, 02H40MZ, 02H432Z, 02H433Z, 02H43DZ, 02H43JZ, 02H43KZ, 02H43MZ, 02H442Z, 02H443Z, 02H44DZ, 02H44JZ, 02H44KZ, 02H44MZ, 02H602Z, 02H603Z, 02H60DZ, 02H60JZ, 02H60KZ, 02H60MZ, 02H632Z, 02H63DZ,	
		02H63JZ, 02H63KZ, 02H63MZ, 02H642Z, 02H643Z, 02H64DZ, 02H64JZ, 02H64KZ, 02H64MZ, 02H702Z, 02H703Z, 02H70DZ, 02H70JZ, 02H70KZ, 02H70MZ, 02H732Z, 02H733Z, 02H73DZ, 02H73JZ, 02H73KZ, 02H73MZ, 02H742Z, 02H743Z, 02H74DZ, 02H74JZ, 02H74KZ, 02H74MZ, 02HA0QZ, 02HA0RS, 02HA0RZ, 02HA3QZ, 02HA3RS, 02HA3RZ, 02HA4QZ, 02HA4RS, 02HA4RZ, 02HK00Z, 02HK02Z, 02HK03Z, 02HK0DZ, 02HK0JZ, 02HK0KZ, 02HK0MZ, 02HK30Z, 02HK32Z, 02HK3DZ, 02HK3JZ, 02HK3KZ, 02HK3MZ, 02HK40Z, 02HK42Z, 02HK43Z, 02HK4DZ, 02HK4JZ, 02HK4KZ, 02HK4MZ, 02HL02Z, 02HL03Z, 02HL0DZ, 02HL0JZ,	

		02HL0KZ, 02HL0MZ, 02HL32Z, 02HL33Z, 02HL3DZ, 02HL3JZ, 02HL3KZ, 02HL3MZ, 02HL42Z, 02HL43Z, 02HL4DZ, 02HL4JZ, 02HL4KZ, 02HL4MZ, 02HN00Z, 02HN02Z, 02HN0JZ, 02HN0KZ, 02HN0MZ, 02HN30Z, 02HN32Z, 02HN3JZ, 02HN3KZ, 02HN3MZ, 02HN40Z, 02HN42Z, 02HN4JZ, 02HN4KZ, 02HN4MZ, 02HW00Z, 02HW30Z, 02HW40Z, 02HX00Z, 02HX30Z, 02HX40Z, 02JA3ZZ, 02JY3ZZ, 02K80ZZ, 02K83ZZ, 02K84ZZ, 02L70CK, 02L70DK, 02L70ZK, 02L73CK, 02L73DK, 02L73ZK, 02L74CK, 02L74DK, 02L74ZK, 02LH0CZ, 02LH0DZ, 02LH0ZZ, 02LH3CZ, 02LH3DZ, 02LH3ZZ, 02LH4CZ, 02LH4DZ, 02LH4ZZ, 02LR0ZT, 02LS0ZZ,	
		02LT0ZZ, 02N40ZZ, 02N43ZZ, 02N44ZZ, 02N50ZZ, 02N53ZZ, 02N54ZZ, 02N60ZZ, 02N63ZZ, 02N64ZZ, 02N70ZZ, 02N73ZZ, 02N74ZZ, 02N80ZZ, 02N83ZZ, 02N84ZZ, 02N90ZZ, 02N93ZZ, 02N94ZZ, 02ND0ZZ, 02ND3ZZ, 02ND4ZZ, 02NF0ZZ, 02NF3ZZ, 02NF4ZZ, 02NG0ZZ, 02NG3ZZ, 02NG4ZZ, 02NH0ZZ, 02NH3ZZ, 02NH4ZZ, 02NJ0ZZ, 02NJ3ZZ, 02NJ4ZZ, 02NK0ZZ, 02NK3ZZ, 02NK4ZZ, 02NL0ZZ, 02NL3ZZ, 02NL4ZZ, 02NM0ZZ, 02NM3ZZ, 02NM4ZZ, 02NN0ZZ, 02NN3ZZ, 02NN4ZZ, 02PA02Z, 02PA03Z, 02PA07Z, 02PA08Z, 02PA0CZ, 02PA0DZ, 02PA0JZ, 02PA0KZ, 02PA0MZ, 02PA0QZ, 02PA0RZ, 02PA32Z, 02PA33Z, 02PA37Z,	
		02PA38Z, 02PA3CZ, 02PA3DZ, 02PA3JZ, 02PA3KZ, 02PA3MZ, 02PA3QZ, 02PA3RZ, 02PA42Z, 02PA43Z, 02PA47Z, 02PA48Z, 02PA4CZ, 02PA4DZ, 02PA4JZ, 02PA4KZ, 02PA4MZ, 02PA4QZ, 02PA4RZ, 02PAXMZ, 02Q00ZZ, 02Q03ZZ, 02Q04ZZ, 02Q10ZZ, 02Q13ZZ, 02Q14ZZ, 02Q20ZZ, 02Q23ZZ, 02Q24ZZ, 02Q30ZZ, 02Q33ZZ, 02Q34ZZ, 02Q40ZZ, 02Q43ZZ, 02Q44ZZ, 02Q50ZZ, 02Q53ZZ, 02Q54ZZ, 02Q60ZZ, 02Q63ZZ, 02Q64ZZ, 02Q70ZZ, 02Q73ZZ, 02Q74ZZ, 02Q80ZZ, 02Q83ZZ, 02Q84ZZ, 02Q90ZZ, 02Q93ZZ, 02Q94ZZ, 02QA0ZZ, 02QA3ZZ, 02QA4ZZ, 02QB0ZZ, 02QB3ZZ, 02QB4ZZ, 02QC0ZZ, 02QC3ZZ, 02QC4ZZ, 02QD0ZZ,	

		02QD3ZZ, 02QD4ZZ, 02QF0ZJ, 02QF0ZZ, 02QF3ZJ, 02QF3ZZ, 02QF4ZJ, 02QF4ZZ, 02QG0ZE, 02QG0ZZ, 02QG3ZE, 02QG3ZZ, 02QG4ZE, 02QG4ZZ, 02QH0ZZ, 02QH3ZZ, 02QH4ZZ, 02QJ0ZG, 02QJ0ZZ, 02QJ3ZG, 02QJ3ZZ, 02QJ4ZG, 02QJ4ZZ, 02QK0ZZ, 02QK3ZZ, 02QK4ZZ, 02QL0ZZ, 02QL3ZZ, 02QL4ZZ, 02QM0ZZ, 02QM3ZZ, 02QM4ZZ, 02QN0ZZ, 02QN3ZZ, 02QN4ZZ, 02R507Z, 02R508Z, 02R50JZ, 02R50KZ, 02R547Z, 02R548Z, 02R54JZ, 02R54KZ, 02R607Z, 02R608Z, 02R60JZ, 02R60KZ, 02R647Z, 02R648Z, 02R64JZ, 02R64KZ, 02R707Z, 02R708Z, 02R70JZ, 02R70KZ, 02R747Z, 02R748Z, 02R74JZ, 02R74KZ, 02R907Z,	
		02R908Z, 02R90JZ, 02R90KZ, 02R947Z, 02R948Z, 02R94JZ, 02R94KZ, 02RD07Z, 02RD08Z, 02RD0JZ, 02RD0KZ, 02RD47Z, 02RD48Z, 02RD4JZ, 02RD4KZ, 02RF07Z, 02RF08Z, 02RF0JZ, 02RF0KZ, 02RF37H, 02RF37Z, 02RF38H, 02RF38Z, 02RF3JH, 02RF3JZ, 02RF3KH, 02RF3KZ, 02RF47Z, 02RF48Z, 02RF4JZ, 02RF4KZ, 02RG07Z, 02RG08Z, 02RG0JZ, 02RG0KZ, 02RG37H, 02RG37Z, 02RG38H, 02RG38Z, 02RG3JH, 02RG3JZ, 02RG3KH, 02RG3KZ, 02RG47Z, 02RG48Z, 02RG4JZ, 02RG4KZ, 02RH07Z, 02RH08Z, 02RH0JZ, 02RH0KZ, 02RH37H, 02RH37Z, 02RH38H, 02RH38Z, 02RH3JH, 02RH3JZ,	
		02RH3KH, 02RH3KZ, 02RH47Z, 02RH48Z, 02RH4JZ, 02RH4KZ, 02RJ07Z, 02RJ08Z, 02RJ0JZ, 02RJ0KZ, 02RJ47Z, 02RJ48Z, 02RJ4JZ, 02RJ4KZ, 02RK07Z, 02RK08Z, 02RK0JZ, 02RK0KZ, 02RK47Z, 02RK48Z, 02RK4JZ, 02RK4KZ, 02RL07Z, 02RL08Z, 02RL0JZ, 02RL0KZ, 02RL47Z, 02RL48Z, 02RL4JZ, 02RL4KZ, 02RM07Z, 02RM08Z, 02RM0JZ, 02RM0KZ, 02RM47Z, 02RM48Z, 02RM4JZ, 02RM4KZ, 02RN07Z, 02RN08Z, 02RN0JZ, 02RN0KZ, 02RN47Z, 02RN48Z, 02RN4JZ, 02RN4KZ, 02RP0JZ, 02RQ07Z, 02RQ0JZ, 02RR07Z, 02RR0JZ, 02S00ZZ, 02S10ZZ, 02SP0ZZ, 02SW0ZZ, 02SX0ZZ, 02T50ZZ, 02T53ZZ, 02T54ZZ, 02T80ZZ, 02T83ZZ, 02T84ZZ, 02T90ZZ, 02T93ZZ, 02T94ZZ, 02TD0ZZ, 02TD3ZZ, 02TD4ZZ,	

		02TH0ZZ, 02TH3ZZ, 02TH4ZZ, 02TM0ZZ, 02TM3ZZ, 02TM4ZZ, 02TN0ZZ, 02TN3ZZ, 02TN4ZZ, 02U507Z, 02U508Z, 02U50JZ, 02U50KZ, 02U537Z, 02U538Z, 02U53JZ, 02U53KZ, 02U547Z, 02U548Z, 02U54JZ, 02U54KZ, 02U607Z, 02U608Z, 02U60JZ, 02U60KZ, 02U637Z, 02U638Z, 02U63JZ, 02U63KZ, 02U647Z, 02U648Z, 02U64JZ, 02U64KZ, 02U707Z, 02U708Z, 02U70JZ, 02U70KZ, 02U737Z, 02U738Z, 02U73JZ, 02U73KZ, 02U747Z, 02U748Z, 02U74JZ, 02U74KZ, 02U907Z, 02U908Z, 02U90JZ, 02U90KZ, 02U937Z, 02U938Z, 02U93JZ, 02U93KZ, 02U947Z, 02U948Z, 02U94JZ, 02U94KZ, 02UA07Z, 02UA08Z, 02UA0JZ, 02UA0KZ, 02UA37Z, 02UA38Z, 02UA3JZ, 02UA3KZ, 02UA47Z, 02UA48Z, 02UA4JZ, 02UA4KZ, 02UD07Z, 02UD08Z, 02UD0JZ, 02UD0KZ, 02UD37Z, 02UD38Z, 02UD3JZ,	
		02UD3KZ, 02UD47Z, 02UD48Z, 02UD4JZ, 02UD4KZ, 02UF07J, 02UF07Z, 02UF08J, 02UF08Z, 02UF0JJ, 02UF0JZ, 02UF0KJ, 02UF0KZ, 02UF37J, 02UF37Z, 02UF38J, 02UF38Z, 02UF3JJ, 02UF3JZ, 02UF3KJ, 02UF3KZ, 02UF47J, 02UF47Z, 02UF48J, 02UF48Z, 02UF4JJ, 02UF4JZ, 02UF4KJ, 02UF4KZ, 02UG07E, 02UG07Z, 02UG08E, 02UG08Z, 02UG0JE, 02UG0JZ, 02UG0KE, 02UG0KZ, 02UG37E, 02UG37Z, 02UG38E, 02UG38Z, 02UG3JE, 02UG3JZ, 02UG3KE, 02UG3KZ, 02UG47E, 02UG47Z, 02UG48E, 02UG48Z, 02UG4JE, 02UG4JZ, 02UG4KE, 02UG4KZ, 02UH07Z, 02UH08Z, 02UH0JZ, 02UH0KZ, 02UH37Z, 02UH38Z, 02UH3JZ, 02UH3KZ, 02UH47Z, 02UH48Z, 02UH4JZ, 02UH4KZ, 02UJ07G, 02UJ07Z, 02UJ08G, 02UJ08Z, 02UJ0JG, 02UJ0JZ, 02UJ0KG, 02UJ0KZ, 02UJ37G, 02UJ37Z, 02UJ38G,	

		02UJ38Z, 02UJ3JG, 02UJ3JZ, 02UJ3KG, 02UJ3KZ, 02UJ47G, 02UJ47Z, 02UJ48G, 02UJ48Z, 02UJ4JG, 02UJ4JZ, 02UJ4KG, 02UJ4KZ, 02UK07Z, 02UK08Z, 02UK0JZ, 02UK0KZ, 02UK37Z, 02UK38Z, 02UK3JZ, 02UK3KZ, 02UK47Z, 02UK48Z, 02UK4JZ, 02UK4KZ, 02UL07Z, 02UL08Z, 02UL0JZ, 02UL0KZ, 02UL37Z, 02UL38Z, 02UL3JZ, 02UL3KZ, 02UL47Z, 02UL48Z, 02UL4JZ, 02UL4KZ, 02UM07Z, 02UM08Z, 02UM0JZ, 02UM0KZ, 02UM37Z, 02UM38Z, 02UM3JZ, 02UM3KZ, 02UM47Z, 02UM48Z, 02UM4JZ, 02UM4KZ, 02UN07Z, 02UN08Z, 02UN0JZ, 02UN0KZ, 02UN37Z, 02UN38Z, 02UN3JZ, 02UN3KZ, 02UN47Z, 02UN48Z, 02UN4JZ, 02UN4KZ, 02VA0CZ, 02VA0ZZ, 02VA3CZ, 02VA3ZZ, 02VA4CZ, 02VA4ZZ, 02VR0ZT, 02W50JZ, 02W54JZ, 02WA02Z, 02WA03Z, 02WA07Z, 02WA08Z, 02WA0CZ, 02WA0DZ, 02WA0JZ, 02WA0KZ, 02WA0MZ, 02WA0QZ, 02WA0RZ, 02WA32Z, 02WA33Z, 02WA37Z, 02WA38Z, 02WA3CZ,	
		02WA3DZ, 02WA3JZ, 02WA3KZ, 02WA3MZ, 02WA3QZ, 02WA3RZ, 02WA42Z, 02WA43Z, 02WA47Z, 02WA48Z, 02WA4CZ, 02WA4DZ, 02WA4JZ, 02WA4KZ, 02WA4MZ, 02WA4QZ, 02WA4RZ, 02WF07Z, 02WF08Z, 02WF0JZ, 02WF0KZ, 02WF47Z, 02WF48Z, 02WF4JZ, 02WF4KZ, 02WG07Z, 02WG08Z, 02WG0JZ, 02WG0KZ, 02WG47Z, 02WG48Z, 02WG4JZ, 02WG4KZ, 02WH07Z, 02WH08Z, 02WH0JZ, 02WH0KZ, 02WH47Z, 02WH48Z, 02WH4JZ, 02WH4KZ, 02WJ07Z, 02WJ08Z, 02WJ0JZ, 02WJ0KZ, 02WJ47Z, 02WJ48Z, 02WJ4JZ, 02WJ4KZ, 02WM0JZ, 02WM4JZ, 02YA0Z0, 02YA0Z1, 02YA0Z2, 0370046, 037004Z, 0370056, 037005Z, 0370066, 037006Z, 0370076, 037007Z,	
		0370346, 037034Z, 0370356, 037035Z, 0370366, 037036Z, 0370376, 037037Z, 0370446, 037044Z, 0370456, 037045Z, 0370466, 037046Z, 0370476, 037047Z, 0371046, 037104Z, 0371056, 037105Z, 0371066, 037106Z, 0371076, 037107Z, 0371346, 037134Z, 0371356, 037135Z, 0371366, 037136Z, 0371376, 037137Z, 0371446, 037144Z, 0371456,	

		037145Z, 0371466, 037146Z, 0371476, 037147Z, 0372046, 037204Z, 0372056, 037205Z, 0372066, 037206Z, 0372076, 037207Z, 0372346, 037234Z, 0372356, 037235Z, 0372366, 037236Z, 0372376, 037237Z, 0372446, 037244Z, 0372456, 037245Z, 0372466, 037246Z, 0372476, 037247Z, 0373046, 037304Z,	
		0373056, 037305Z, 0373066, 037306Z, 0373076, 037307Z, 0373346, 037334Z, 0373356, 037335Z, 0373366, 037336Z, 0373376, 037337Z, 0373446, 037344Z, 0373456, 037345Z, 0373466, 037346Z, 0373476, 037347Z, 0374046, 037404Z, 0374056, 037405Z, 0374066, 037406Z, 0374076, 037407Z, 0374346, 037434Z, 0374356, 037435Z, 0374366, 037436Z, 0374376, 037437Z, 0374446, 037444Z, 0374456, 037445Z, 0374466, 037446Z, 0374476, 037447Z, 0375046, 037504Z, 0375056, 037505Z, 0375066, 037506Z, 0375076, 037507Z, 0375346, 037534Z, 0375356, 037535Z, 0375366, 037536Z, 0375376, 037537Z, 0375446, 037544Z, 0375456, 037545Z, 0375466,	
		037546Z, 0375476, 037547Z, 0376046, 037604Z, 0376056, 037605Z, 0376066, 037606Z, 0376076, 037607Z, 0376346, 037634Z, 0376356, 037635Z, 0376366, 037636Z, 0376376, 037637Z, 0376446, 037644Z, 0376456, 037645Z, 0376466, 037646Z, 0376476, 037647Z, 0377046, 037704Z, 0377056, 037705Z, 0377066, 037706Z, 0377076, 037707Z, 0377346, 037734Z, 0377356, 037735Z, 0377366, 037736Z, 0377376, 037737Z, 0377446, 037744Z, 0377456, 037745Z, 0377466, 037746Z, 0377476, 037747Z, 0378046, 037804Z, 0378056, 037805Z, 0378066, 037806Z, 0378076, 037807Z, 0378346, 037834Z, 0378356, 037835Z, 0378366, 037836Z, 0378376, 037837Z, 0378446, 037844Z, 0378456, 037845Z, 0378466, 037846Z, 0378476, 037847Z, 0379046, 037904Z, 0379056,	

		037905Z, 0379066, 037906Z, 0379076, 037907Z, 0379346, 037934Z, 0379356, 037935Z, 0379366, 037936Z, 0379376, 037937Z, 0379446, 037944Z, 0379456, 037945Z, 0379466, 037946Z, 0379476, 037947Z, 037A046, 037A04Z, 037A056, 037A05Z, 037A066, 037A06Z, 037A076, 037A07Z, 037A346, 037A34Z, 037A356, 037A35Z, 037A366, 037A36Z, 037A376, 037A37Z, 037A446, 037A44Z, 037A456, 037A45Z, 037A466, 037A46Z, 037A476, 037A47Z, 037B046, 037B04Z, 037B056, 037B05Z, 037B066, 037B06Z, 037B076, 037B07Z, 037B346, 037B34Z, 037B356, 037B35Z, 037B366, 037B36Z, 037B376, 037B37Z, 037B446, 037B44Z, 037B456, 037B45Z, 037B466, 037B46Z,	
		037B476, 037B47Z, 037C046, 037C04Z, 037C056, 037C05Z, 037C066, 037C06Z, 037C076, 037C07Z, 037C346, 037C34Z, 037C356, 037C35Z, 037C366, 037C36Z, 037C376, 037C37Z, 037C446, 037C44Z, 037C456, 037C45Z, 037C466, 037C46Z, 037C476, 037C47Z, 037D046, 037D04Z, 037D056, 037D05Z, 037D066, 037D06Z, 037D076, 037D07Z, 037D346, 037D34Z, 037D356, 037D35Z, 037D366, 037D36Z, 037D376, 037D37Z, 037D446, 037D44Z, 037D456, 037D45Z, 037D466, 037D46Z, 037D476, 037D47Z, 037F046, 037F04Z, 037F056, 037F05Z, 037F066, 037F06Z, 037F076, 037F07Z, 037F346, 037F34Z, 037F356, 037F35Z, 037F366, 037F36Z, 037F376, 037F37Z,	
		037F446, 037F44Z, 037F456, 037F45Z, 037F466, 037F46Z, 037F476, 037F47Z, 037G046, 037G04Z, 037G056, 037G05Z, 037G066, 037G06Z, 037G076, 037G07Z, 037H046, 037H04Z, 037H056, 037H05Z, 037H066, 037H06Z, 037H076, 037H07Z, 037H346, 037H34Z, 037H356, 037H35Z, 037H366, 037H36Z, 037H376, 037H37Z, 037H446, 037H44Z, 037H456, 037H45Z, 037H466, 037H46Z, 037H476, 037H47Z, 037J046, 037J04Z, 037J056, 037J05Z, 037J066, 037J06Z, 037J076, 037J07Z, 037J346, 037J34Z,	

		037J356, 037J35Z, 037J366, 037J36Z, 037J376, 037J37Z, 037J446, 037J44Z, 037J456, 037J45Z, 037J466, 037J46Z, 037J476, 037J47Z, 037K046, 037K04Z, 037K056, 037K05Z,	
		037K066, 037K06Z, 037K076, 037K07Z, 037K346, 037K34Z, 037K356, 037K35Z, 037K366, 037K36Z, 037K376, 037K37Z, 037K446, 037K44Z, 037K456, 037K45Z, 037K466, 037K46Z, 037K476, 037K47Z, 037L046, 037L04Z, 037L056, 037L05Z, 037L066, 037L06Z, 037L076, 037L07Z, 037L346, 037L34Z, 037L356, 037L35Z, 037L366, 037L36Z, 037L376, 037L37Z, 037L446, 037L44Z, 037L456, 037L45Z, 037L466, 037L46Z, 037L476, 037L47Z, 037M046, 037M04Z, 037M056, 037M05Z, 037M066, 037M06Z, 037M076, 037M07Z, 037M346, 037M34Z, 037M356, 037M35Z, 037M366, 037M36Z, 037M376, 037M37Z, 037M446, 037M44Z, 037M456, 037M45Z, 037M466,	
		037M46Z, 037M476, 037M47Z, 037N046, 037N04Z, 037N056, 037N05Z, 037N066, 037N06Z, 037N076, 037N07Z, 037N346, 037N34Z, 037N356, 037N35Z, 037N366, 037N36Z, 037N376, 037N37Z, 037N446, 037N44Z, 037N456, 037N45Z, 037N466, 037N46Z, 037N476, 037N47Z, 037P046, 037P04Z, 037P056, 037P05Z, 037P066, 037P06Z, 037P076, 037P07Z, 037P346, 037P34Z, 037P356, 037P35Z, 037P366, 037P36Z, 037P376, 037P37Z, 037P446, 037P44Z, 037P456, 037P45Z, 037P466, 037P46Z, 037P476, 037P47Z, 037Q046, 037Q04Z, 037Q056, 037Q05Z, 037Q066, 037Q06Z, 037Q076, 037Q07Z, 037Q346, 037Q34Z, 037Q356, 037Q35Z, 037Q366, 037Q36Z, 037Q376,	

		037Q37Z, 037Q446, 037Q44Z, 037Q456, 037Q45Z, 037Q466, 037Q46Z, 037Q476, 037Q47Z, 037R046, 037R04Z, 037R056, 037R05Z, 037R066, 037R06Z, 037R076, 037R07Z, 037R346, 037R34Z, 037R356, 037R35Z, 037R366, 037R36Z, 037R376, 037R37Z, 037R446, 037R44Z, 037R456, 037R45Z, 037R466, 037R46Z, 037R476, 037R47Z, 037S046, 037S04Z, 037S056, 037S05Z, 037S066, 037S06Z, 037S076, 037S07Z, 037S346, 037S34Z, 037S356, 037S35Z, 037S366, 037S36Z, 037S376, 037S37Z, 037S446, 037S44Z, 037S456, 037S45Z, 037S466, 037S46Z, 037S476,	
		037S47Z, 037T046, 037T04Z, 037T056, 037T05Z, 037T066, 037T06Z, 037T076, 037T07Z, 037T346, 037T34Z, 037T356, 037T35Z, 037T366, 037T36Z, 037T376, 037T37Z, 037T446, 037T44Z, 037T456, 037T45Z, 037T466, 037T46Z, 037T476, 037T47Z, 037U046, 037U04Z, 037U056, 037U05Z, 037U066, 037U06Z, 037U076, 037U07Z, 037U346, 037U34Z, 037U356, 037U35Z, 037U366, 037U36Z, 037U376, 037U37Z, 037U446, 037U44Z, 037U456, 037U45Z, 037U466, 037U46Z, 037U476, 037U47Z, 037V046, 037V04Z, 037V056, 037V05Z, 037V066, 037V06Z, 037V076, 037V07Z, 037V346, 037V34Z, 037V356, 037V35Z, 037V366, 037V36Z, 037V376, 037V37Z, 037V446,	
		037V44Z, 037V456, 037V45Z, 037V466, 037V46Z, 037V476, 037V47Z, 037Y046, 037Y04Z, 037Y056, 037Y05Z, 037Y066, 037Y06Z, 037Y076, 037Y07Z, 037Y346, 037Y34Z, 037Y356, 037Y35Z, 037Y366, 037Y36Z, 037Y376, 037Y37Z, 037Y446, 037Y44Z, 037Y456, 037Y45Z, 037Y466, 037Y46Z, 037Y476, 037Y47Z, 0470046, 047004Z, 0470056, 047005Z, 0470066, 047006Z, 0470076, 047007Z, 0470346, 047034Z, 0470356, 047035Z, 0470366, 047036Z, 0470376, 047037Z, 0470446, 047044Z, 0470456, 047045Z, 0470466, 047046Z, 0470476, 047047Z, 0471046, 047104Z, 0471056, 047105Z, 0471066,	

		047106Z, 0471076, 047107Z, 0471346, 047134Z, 0471356,	
		047135Z, 0471366, 047136Z, 0471376, 047137Z, 0471446, 047144Z, 0471456, 047145Z, 0471466, 047146Z, 0471476, 047147Z, 0472046, 047204Z, 0472056, 047205Z, 0472066, 047206Z, 0472076, 047207Z, 0472346, 047234Z, 0472356, 047235Z, 0472366, 047236Z, 0472376, 047237Z, 0472446, 047244Z, 0472456, 047245Z, 0472466, 047246Z, 0472476, 047247Z, 0473046, 047304Z, 0473056, 047305Z, 0473066, 047306Z, 0473076, 047307Z, 0473346, 047334Z, 0473356, 047335Z, 0473366, 047336Z, 0473376, 047337Z, 0473446, 047344Z, 0473456, 047345Z, 0473466, 047346Z, 0473476, 047347Z, 0474046, 047404Z, 0474056, 047405Z, 0474066, 047406Z, 0474076,	

		047407Z, 0474346, 047434Z, 0474356, 047435Z, 0474366, 047436Z, 0474376, 047437Z, 0474446, 047444Z, 0474456, 047445Z, 0474466, 047446Z, 0474476, 047447Z, 0475046, 047504Z, 0475056, 047505Z, 0475066, 047506Z, 0475076, 047507Z, 0475346, 047534Z, 0475356, 047535Z, 0475366, 047536Z, 0475376, 047537Z, 0475446, 047544Z, 0475456, 047545Z, 0475466, 047546Z, 0475476, 047547Z, 0476046, 047604Z, 0476056, 047605Z, 0476066, 047606Z, 0476076, 047607Z, 0476346, 047634Z, 0476356, 047635Z, 0476366, 047636Z, 0476376, 047637Z, 0476446, 047644Z, 0476456, 047645Z, 0476466, 047646Z, 0476476, 047647Z, 0477046, 047704Z, 0477056, 047705Z, 0477066, 047706Z, 0477076, 047707Z, 0477346, 047734Z, 0477356, 047735Z, 0477366,	
		047736Z, 0477376, 047737Z, 0477446, 047744Z, 0477456, 047745Z, 0477466, 047746Z, 0477476, 047747Z, 0478046, 047804Z, 0478056, 047805Z, 0478066, 047806Z, 0478076, 047807Z, 0478346, 047834Z, 0478356, 047835Z, 0478366, 047836Z, 0478376, 047837Z, 0478446, 047844Z, 0478456, 047845Z, 0478466, 047846Z, 0478476, 047847Z, 0479046, 047904Z, 0479056, 047905Z, 0479066, 047906Z, 0479076, 047907Z, 0479346, 047934Z, 0479356, 047935Z, 0479366, 047936Z, 0479376, 047937Z, 0479446, 047944Z, 0479456, 047945Z, 0479466, 047946Z, 0479476, 047947Z, 047A046, 047A04Z, 047A056, 047A05Z, 047A066, 047A06Z, 047A076, 047A07Z, 047A346, 047A34Z, 047A356, 047A35Z, 047A366, 047A36Z, 047A376, 047A37Z, 047A446, 047A44Z,	
		047A456, 047A45Z, 047A466, 047A46Z, 047A476, 047A47Z, 047B046, 047B04Z, 047B056, 047B05Z, 047B066, 047B06Z, 047B076, 047B07Z, 047B346, 047B34Z, 047B356, 047B35Z, 047B366, 047B36Z, 047B376, 047B37Z, 047B446, 047B44Z, 047B456, 047B45Z, 047B466, 047B46Z, 047B476, 047B47Z,	

		047C046, 047C04Z, 047C056, 047C05Z, 047C066, 047C06Z, 047C076, 047C07Z, 047C346, 047C34Z, 047C356, 047C35Z, 047C366, 047C36Z, 047C376, 047C37Z, 047C446, 047C44Z, 047C456, 047C45Z, 047C466, 047C46Z, 047C476, 047C47Z, 047D046, 047D04Z, 047D056, 047D05Z, 047D066, 047D06Z, 047D076, 047D07Z, 047D346, 047D34Z, 047D356, 047D35Z, 047D366,	
		047D36Z, 047D376, 047D37Z, 047D446, 047D44Z, 047D456, 047D45Z, 047D466, 047D46Z, 047D476, 047D47Z, 047E046, 047E04Z, 047E056, 047E05Z, 047E066, 047E06Z, 047E076, 047E07Z, 047E346, 047E34Z, 047E356, 047E35Z, 047E366, 047E36Z, 047E376, 047E37Z, 047E446, 047E44Z, 047E456, 047E45Z, 047E466, 047E46Z, 047E476, 047E47Z, 047F046, 047F04Z, 047F056, 047F05Z, 047F066, 047F06Z, 047F076, 047F07Z, 047F346, 047F34Z, 047F356, 047F35Z, 047F366, 047F36Z, 047F376, 047F37Z, 047F446, 047F44Z, 047F456, 047F45Z, 047F466, 047F46Z, 047F476, 047F47Z, 047H046, 047H04Z, 047H056, 047H05Z, 047H066, 047H06Z, 047H076, 047H07Z, 047H346, 047H34Z, 047H356, 047H35Z, 047H366, 047H36Z, 047H376, 047H37Z, 047H446,	
		047H44Z, 047H456, 047H45Z, 047H466, 047H46Z, 047H476, 047H47Z, 047J046, 047J04Z, 047J056, 047J05Z, 047J066, 047J06Z, 047J076, 047J07Z, 047J346, 047J34Z, 047J356, 047J35Z, 047J366, 047J36Z, 047J376, 047J37Z, 047J446, 047J44Z, 047J456, 047J45Z, 047J466, 047J46Z, 047J476, 047J47Z, 047K041, 047K046, 047K04Z, 047K056, 047K05Z, 047K066, 047K06Z, 047K076, 047K07Z, 047K341, 047K346, 047K34Z, 047K356, 047K35Z, 047K366, 047K36Z, 047K376, 047K37Z, 047K441, 047K446, 047K44Z, 047K456, 047K45Z, 047K466, 047K46Z, 047K476, 047K47Z, 047L041, 047L046, 047L04Z, 047L056, 047L05Z, 047L066, 047L06Z, 047L076, 047L07Z, 047L341, 047L346, 047L34Z,	

		047L356, 047L35Z, 047L366, 047L36Z, 047L376, 047L37Z, 047L441, 047L446, 047L44Z, 047L456, 047L45Z, 047L466, 047L46Z, 047L476, 047L47Z, 047M041, 047M046, 047M04Z, 047M056, 047M05Z, 047M066, 047M06Z, 047M076, 047M07Z, 047M341, 047M346, 047M34Z, 047M356, 047M35Z, 047M366, 047M36Z, 047M376, 047M37Z, 047M441, 047M446, 047M44Z, 047M456, 047M45Z, 047M466, 047M46Z, 047M476, 047M47Z, 047N041, 047N046, 047N04Z, 047N056, 047N05Z, 047N066, 047N06Z, 047N076, 047N07Z, 047N341, 047N346, 047N34Z, 047N356, 047N35Z, 047N366, 047N36Z, 047N376, 047N37Z, 047N441, 047N446, 047N44Z, 047N456,	
		047N45Z, 047N466, 047N46Z, 047N476, 047N47Z, 047P046, 047P04Z, 047P056, 047P05Z, 047P066, 047P06Z, 047P076, 047P07Z, 047P346, 047P34Z, 047P356, 047P35Z, 047P366, 047P36Z, 047P376, 047P37Z, 047P446, 047P44Z, 047P456, 047P45Z, 047P466, 047P46Z, 047P476, 047P47Z, 047Q046, 047Q04Z, 047Q056, 047Q05Z, 047Q066, 047Q06Z, 047Q076, 047Q07Z, 047Q346, 047Q34Z, 047Q356, 047Q35Z, 047Q366, 047Q36Z, 047Q376, 047Q37Z, 047Q446, 047Q44Z, 047Q456, 047Q45Z, 047Q466, 047Q46Z, 047Q476, 047Q47Z, 047R046, 047R04Z, 047R056, 047R05Z, 047R066, 047R06Z, 047R076, 047R07Z, 047R346, 047R34Z, 047R356, 047R35Z, 047R366, 047R36Z, 047R376, 047R37Z, 047R446, 047R44Z, 047R456, 047R45Z, 047R466, 047R46Z, 047R476, 047R47Z,	

		047S046, 047S04Z, 047S056, 047S05Z, 047S066, 047S06Z, 047S076, 047S07Z, 047S346, 047S34Z, 047S356, 047S35Z, 047S366, 047S36Z, 047S376, 047S37Z, 047S446, 047S44Z, 047S456, 047S45Z, 047S466, 047S46Z, 047S476, 047S47Z, 047T046, 047T04Z, 047T056, 047T05Z, 047T066, 047T06Z, 047T076, 047T07Z, 047T346, 047T34Z, 047T356, 047T35Z, 047T366, 047T36Z, 047T376, 047T37Z, 047T446, 047T44Z, 047T456, 047T45Z, 047T466, 047T46Z, 047T476, 047T47Z, 047U046, 047U04Z, 047U056, 047U05Z, 047U066, 047U06Z, 047U076, 047U07Z, 047U346, 047U34Z, 047U356, 047U35Z, 047U366, 047U36Z, 047U376, 047U37Z, 047U446, 047U44Z, 047U456, 047U45Z, 047U466, 047U46Z, 047U476, 047U47Z, 047V046, 047V04Z, 047V056, 047V05Z, 047V066, 047V06Z, 047V076, 047V07Z, 047V346, 047V34Z, 047V356, 047V35Z, 047V366, 047V36Z, 047V376, 047V37Z,	
		047V446, 047V44Z, 047V456, 047V45Z, 047V466, 047V46Z, 047V476, 047V47Z, 047W046, 047W04Z, 047W056, 047W05Z, 047W066, 047W06Z, 047W076, 047W07Z, 047W346, 047W34Z, 047W356, 047W35Z, 047W366, 047W36Z, 047W376, 047W37Z, 047W446, 047W44Z, 047W456, 047W45Z, 047W466, 047W46Z, 047W476, 047W47Z, 047Y046, 047Y04Z, 047Y056, 047Y05Z, 047Y066, 047Y06Z, 047Y076, 047Y07Z, 047Y346, 047Y34Z, 047Y356, 047Y35Z, 047Y366, 047Y36Z, 047Y376, 047Y37Z, 047Y446, 047Y44Z, 047Y456, 047Y45Z, 047Y466, 047Y46Z, 047Y476, 047Y47Z, 04H002Z, 04H032Z, 04H042Z, 0JH600Z, 0JH602Z, 0JH604Z, 0JH605Z, 0JH606Z, 0JH607Z,	

		0JH608Z, 0JH609Z, 0JH60PZ, 0JH630Z, 0JH632Z, 0JH634Z, 0JH635Z, 0JH636Z, 0JH637Z, 0JH638Z, 0JH639Z, 0JH63PZ, 0JH800Z, 0JH804Z, 0JH805Z, 0JH806Z, 0JH807Z, 0JH808Z, 0JH809Z, 0JH80PZ, 0JH830Z, 0JH834Z, 0JH835Z, 0JH836Z, 0JH837Z, 0JH838Z, 0JH839Z, 0JH83PZ, 0JPT0PZ, 0JPT3PZ, 0JWT02Z, 0JWT0PZ, 0JWT32Z, 0JWT3PZ, 0W9D00Z, 0W9D0ZX, 0W9D0ZZ, 0W9D30Z, 0W9D3ZX, 0W9D3ZZ, 0W9D40Z, 0W9D4ZX, 0W9D4ZZ, 0WCD0ZZ, 0WCD3ZZ, 0WCD4ZZ, 0WFD0ZZ, 0WFD3ZZ, 0WFD4ZZ, 0WFDXZZ, 0WHD03Z, 0WHD0YZ, 0WHD33Z, 0WHD3YZ, 0WHD43Z, 0WHD4YZ, 0WJD0ZZ, 0WJD3ZZ, 0WPD00Z, 0WPD01Z, 0WPD03Z, 0WPD0YZ, 0WPD30Z, 0WPD31Z,	
		0WPD33Z, 0WPD3YZ, 0WPD40Z, 0WPD41Z, 0WPD43Z, 0WPD4YZ, 0WWD00Z, 0WWD01Z, 0WWD03Z, 0WWD0YZ, 0WWD30Z, 0WWD31Z, 0WWD33Z, 0WWD3YZ, 0WWD40Z, 0WWD41Z, 0WWD43Z, 0WWD4YZ, 3E053KZ, 3E063KZ, 3E07017, 3E070GC, 3E070KZ, 3E070PZ, 3E07317, 3E073GC, 3E073KZ, 3E073PZ, 3E080GC, 3E080KZ, 3E083GC, 3E083KZ, 4A020N6, 4A020N7, 4A020N8, 4A023FZ, 4A023N6, 4A023N7, 4A023N8, 4A02X4Z, 4A02XFZ, 4A030BC, 4A033BC, 4A130BC, 4A133BC, 5A02110, 5A02116, 5A0211D, 5A02210, 5A02216, 5A0221D, 5A1213Z, 5A1223Z, B244YZZ, B244ZZZ, B245YZZ, B245ZZZ, B246YZZ, B246ZZZ, B24DYZZ, B24DZZZ, X2RF032, X2RF332, X2RF432	

CPT, current procedural terminology.

Table 3S. Outcome ICD-9 and ICD-10 codes.

Medical conditions	ICD-9 codes	Description	ICD-10 codes
Stroke		Inclusive of ischemic stroke and hemorrhagic stroke below	
Major bleeding		Inclusive of hemorrhagic stroke, major intracranial bleeding and major extracranial bleeding below	
Ischemic stroke (primary only)	433.x1	Occlusion and stenosis of pre-cerebral arteries with cerebral infarction	I63.xxx
	434.x1	Occlusion of cerebral arteries with cerebral infarction	
	436	Acute, but ill-defined, cerebrovascular disease	I67.89
	Exclusion	EXCLUDE above codes if hospitalization lasted <48 hours and was accompanied by carotid endarterectomy (ICD-9 procedure code 38.1)	
Hemorrhagic stroke (primary only)	431	Intracerebral hemorrhage (ICH)	I61.x
	Exclusion	EXCLUDE above codes if "traumatic brain injury" ICD-9-CM code (800 to804, 850 to 854) or "rehabilitation care" as the primary ICD-9-CM code (V57) is present	S02.xxxA, S02.xxxB, S06.xxxA, Z51.89
Major intracranial bleeding (primary or secondary)	430	Subarachnoid hemorrhage	I60.xx
	431	Intracerebral hemorrhage	I61.x
	432.x	Other and unspecified intracranial hemorrhage	I62.xx
	852.0	Subarachnoid hemorrhage following injury without mention of open intracranial wound	S06.36xA
	852.2	Subdural hemorrhage following injury without mention of open intracranial wound	S06.4XxA

	852.4	Extradural hemorrhage following injury without mention of open intracranial wound	S06.5XxA
	853.0	Other and unspecified intracranial hemorrhage following injury without mention of open intracranial wound	S06.6XxA
	Exclusion	EXCLUDE – above codes if concomitant discharge diagnosis of major trauma was present (*ICD-9* codes 852.1, 852.3, 852.5, and 853.1)	S01.90XA, S06.36xA, S06.4XxA, S06.5XxA, S06.6XxA
Major extracranial		Inclusive of major GI bleeding, major urogenital bleeding and major other bleeding	
Major GI bleeding		Inclusive of major upper GI bleeding and major lower GI bleeding	
Major upper GI bleed (primary only)	531.0x	Acute gastric ulcer with hemorrhage with/without obstruction	K25.0
	531.2x	With hemorrhage and perforation with/without obstruction	K25.2
	531.4x	(chronic or unspecified gastric ulcer with hemorrhage with/without obstruction)	K25.4
	531.6x	(with hemorrhage and perforation with/without obstruction)	K25.6
	532.0x	(acute duodenal ulcer with hemorrhage with/without obstruction)	K26.0
	532.2x	(with hemorrhage and perforation with/without obstruction)	K26.2
	532.4x	(chronic or unspecified duodenal ulcer with hemorrhage with/without obstruction)	K26.4

	532.6x	(with hemorrhage and perforation with/without obstruction)	K26.6
	533.0x	(acute peptic ulcer of unspecified site with hemorrhage with/without obstruction)	K27.0
	533.2x	(with hemorrhage and perforation with/without obstruction)	K27.2
	533.4x	(chronic or unspecified peptic ulcer of unspecified site with hemorrhage with/without obstruction)	K27.4
	533.6x	(with hemorrhage and perforation with/without obstruction),	K27.6
	534.0x	(acute gastrojejunal ulcer with hemorrhage with/without obstruction)	K28.0
	534.2x	(with hemorrhage and perforation with/without obstruction)	K28.2
	534.4x	(chronic or unspecified gastrojejunal ulcer with hemorrhage with/without obstruction)	K28.4
	534.6x	(with hemorrhage and perforation with/without obstruction)	K28.6
	578.0	(hematemesis)	K92.0
	ICD-9 procedure code 44.43	(endoscopic control of gastric or duodenal bleeding)	
	CPT code 43255	(upper gastrointestinal endoscopy including esophagus, stomach, and either the duodenum and/or jejunum as appropriate with control of bleeding, any method)	
Major lower GI bleeding (primary only)	562.02	Diverticulosis of small intestine with hemorrhage	K55.21
	562.03	Diverticulitis of small intestine with hemorrhage	K57.11

	562.12	Diverticulosis of colon with hemorrhage	K57.13
	562.13	Diverticulitis of colon with hemorrhage	K57.31
	569.3x	Hemorrhage of rectum and anus	K57.32
	569.85	Angiodysplasia of intestine with hemorrhage	K62.5
	578.1x	Blood in stool	K92.1
	578.9	Hemorrhage of GI tract, unspecified	K92.2
Major urogenital bleed (primary only)	599.7	Hematuria	R31.0 - R31.29
	626.2x and (280.0, 285.1 or 285.9)	Excessive/frequent menstruation and secondary diagnosis indicating acute bleeding (anemia)	N92.0 and (D50.0, D62, D64.9)
Other major bleeds (primary only)	719.1x	Hemathrosis	D62
	423.0x	Hemopericardium	I31.2
	786.3x	Hemoptysis	M25.0xx
	784.7x	Epistaxis	R04.0
	459.0x	Hemorrhage not specified	R04.2
	285.1x	Acute posthemorrhagic anemia	R04.8x, R04.9, R58
TIA (primary only)	435.x	Transient cerebral ischemia as the principal (primary) discharge diagnosis	G45.0, G45.1, G45.8, G45.9, G46.0, G46.1, G46.2
Myocardial infarction (primary position only)	410.xx	Acute myocardial infarction, excluding 410.x2	I21.xx (excluding I21.09, I21.19, I21.11, I21.29, I21.3, I21.4)
VTE		Inclusive of DVT and PE	
	451.xx	Phlebitis and thrombophlebitis	I80.xxx

DVT (primary or secondary only)	453.xx	Other venous embolism and thrombosis	I82.xxx
PE (Primary or secondary only)	415.1x	Pulmonary embolism	I26.01, I26.09, I26.90, I26.92, I26.99, I27.82

Table 4S. Balance assessment: dabigatran versus rivaroxaban.

	Pre-match			Post-match		
**Variable description**	**Dabigatran**	**Rivaroxaban**	**STD**	**Dabigatran**	**Rivaroxaban**	**STD**
Age (mean)	70.89	71.28	0.04	70.89	70.92	0.00
Sex, female (P)	0.38	0.40	0.03	0.38	0.39	0.01
Race (P)			0.04			0.00
White	0.30	0.31		0.30	0.30	
Black	0.02	0.02		0.02	0.02	
Other/Unknown/Missing	0.68	0.67		0.68	0.68	
Region (census) (P)			0.02			0.00
Northeast	0.07	0.07		0.07	0.07	
Midwest	0.12	0.11		0.12	0.12	
South	0.58	0.59		0.58	0.58	
West	0.23	0.22		0.23	0.23	
Missing	0.01	0.01		0.01	0.01	
Diagnosis on the index date or during the 1-year pre-index period (P)						
Cancer	0.10	0.10	0.00	0.10	0.10	0.00
Rheumatoid arthritis	0.02	0.02	0.01	0.02	0.02	0.00
Coronary artery disease	0.31	0.31	0.01	0.31	0.31	0.00
Acute myocardial infarction	0.02	0.02	0.02	0.02	0.02	0.00

Cardiomyopathy	0.06	0.06	0.01	0.06	0.06	0.00
Ischemic stroke	0.06	0.06	0.01	0.06	0.06	0.00
Stroke (all types)	0.08	0.08	0.01	0.08	0.08	0.00
TIA	0.05	0.05	0.00	0.05	0.05	0.01
CHF	0.14	0.14	0.01	0.14	0.14	0.00
Left ventricular disease	0.00	0.00	0.02	0.00	0.00	-
Hypertension	0.75	0.75	0.01	0.75	0.75	0.00
Peripheral artery disease	0.09	0.09	0.01	0.09	0.09	0.00
Liver disease	0.03	0.05	0.10	0.03	0.03	0.01
Renal disease	0.15	0.14	0.03	0.15	0.14	0.01
COPD	0.17	0.17	0.01	0.17	0.17	0.00
Diabetes	0.28	0.27	0.01	0.28	0.28	0.01
Peptic ulcer/GERD	0.20	0.21	0.03	0.20	0.19	0.01
Venous disease	0.01	0.00	0.01	0.01	0.00	0.01
Hyperlipidemia	0.00	0.01	0.06	0.00	0.00	0.00
HIV	0.00	0.00	0.01	0.00	0.00	0.00
Bone marrow disease	0.02	0.02	0.03	0.02	0.02	0.00
Coagulopathy	0.00	0.00	0.02	0.00	0.00	0.00
Chronic kidney disease	0.07	0.06	0.03	0.07	0.07	0.25
Prescription on the index date or during the 1-year pre-index period (P)						
Heparin	0.01	0.01	0.04	0.01	0.01	1.42

Low molecular weight heparins	0.01	0.01	0.01	0.01	0.01	0.01
Beta blockers	0.53	0.53	0.00	0.53	0.53	0.00
Calcium channel	0.32	0.31	0.02	0.32	0.32	0.00
Diuretics	0.35	0.33	0.04	0.35	0.35	0.00
Antihypertensives	0.59	0.59	0.02	0.59	0.60	0.00
Antihyperlipidemics	0.56	0.58	0.05	0.56	0.56	0.00
Corticosteroids	0.20	0.22	0.05	0.20	0.19	0.00
Antidiabetics	0.22	0.22	0.01	0.22	0.21	0.01
Antiarrhythmic	0.17	0.16	0.03	0.17	0.17	0.00
Ketoconazole	0.00	0.00	0.01	0.00	0.00	0.00
Antiplatelet	0.13	0.13	0.00	0.13	0.13	0.00
NSAIDs	0.25	0.25	0.01	0.25	0.24	0.00
Risk score on the index date or during the 1-year pre-index period (mean)						
CCI score	4.27	4.30	0.01	4.27	4.26	0.00
$CHADS_2$ score	1.77	1.77	0.00	1.77	1.77	0.00
CHA_2DS_2-VASc score	3.10	3.13	0.02	3.10	3.10	0.00
HAS-BLED score	2.32	2.34	0.01	2.32	2.32	0.01

Race categories were White, Black or Other/Unknown/Missing.

Region categories were US census regions: Northeast, Midwest, South, West, or Missing.

CCI, Charlson comorbidity index; CHF, congestive heart failure; COPD, chronic objective pulmonary disease; GERD, Gastroesophageal reflux disease; HIV, human immunodeficiency virus; NSAID, non-steroidal anti-inflammatory drug; P, proportion; STD, standardized difference; TIA, transient ischemic attack.

Table 5S. Balance assessment: dabigatran versus apixaban.

	Pre-match			Post-match		
**Variable description**	**Dabigatran**	**Apixaban**	**STD**	**Dabigatran**	**Apixaban**	**STD**
Age (mean)	70.15	72.35	0.23	70.15	70.20	0.01
Sex, female (P)	0.37	0.40	0.07	0.37	0.37	0.00
Race (P)			0.10			0.00
White	0.34	0.30		0.34	0.34	
Black	0.03	0.02		0.03	0.03	
Other/Unknown/Missing	0.63	0.68		0.63	0.63	
Region (census) (P)			0.01			0.02
Northeast	0.07	0.06		0.07	0.07	
Midwest	0.13	0.11		0.13	0.13	
South	0.55	0.62		0.55	0.55	
West	0.23	0.20		0.23	0.24	
Missing	0.01	0.01		0.01	0.01	
Diagnosis on the index date or during the 1-year pre-index period (P)						
Cancer	0.09	0.12	0.07	0.09	0.09	0.01
Rheumatoid arthritis	0.02	0.02	0.01	0.02	0.02	0.00
Coronary artery disease	0.29	0.33	0.10	0.29	0.28	0.01
Acute myocardial infarction	0.02	0.03	0.04	0.02	0.02	0.01

Cardiomyopathy	0.06	0.07	0.02	0.06	0.06	0.01
Ischemic stroke	0.06	0.07	0.05	0.06	0.05	0.01
Stroke (all types)	0.07	0.08	0.05	0.07	0.07	0.00
TIA	0.04	0.05	0.03	0.04	0.04	0.01
CHF	0.14	0.16	0.07	0.14	0.14	0.00
Left ventricular disease	0.00	0.00	0.02	0.00	0.00	-
Hypertension	0.73	0.77	0.10	0.73	0.73	0.01
Peripheral artery disease	0.08	0.10	0.06	0.08	0.09	0.02
Liver disease	0.05	0.10	0.17	0.05	0.05	0.00
Renal disease	0.15	0.18	0.08	0.15	0.15	0.01
COPD	0.17	0.17	0.01	0.17	0.17	0.01
Diabetes	0.28	0.29	0.01	0.28	0.28	0.01
Peptic ulcer/GERD	0.19	0.23	0.10	0.19	0.19	0.00
Venous disease	0.01	0.00	0.01	0.01	0.01	0.00
Hyperlipidemia	0.01	0.03	0.12	0.01	0.01	0.02
HIV	0.00	0.00	0.01	0.00	0.00	0.01
Bone marrow disease	0.02	0.03	0.06	0.02	0.02	0.01
Coagulopathy	0.00	0.00	0.02	0.00	0.00	0.00
Chronic kidney disease	0.07	0.10	0.10	0.07	0.07	0.00
Prescription on the index date or during the 1-year pre-index period (P)						
Heparin	0.01	0.01	0.02	0.01	0.01	0.00

Low molecular weight heparins	0.01	0.01	0.02	0.01	0.01	0.01
Beta blockers	0.53	0.57	0.09	0.53	0.52	0.01
Calcium channel	0.31	0.34	-6.12	0.31	0.31	0.00
Diuretics	0.34	0.35	-2.78	0.34	0.33	0.01
Antihypertensives	0.58	0.61	-5.13	0.58	0.58	0.00
Antihyperlipidemics	0.58	0.62	-7.84	0.58	0.58	0.01
Corticosteroids	0.20	0.22	-4.19	0.20	0.20	0.00
Antidiabetics	0.22	0.23	-0.93	0.22	0.22	0.00
Antiarrhythmic	0.17	0.18	-3.27	0.17	0.17	0.01
Ketoconazole	0.00	0.00	-0.45	0.00	0.00	0.01
Antiplatelet	0.11	0.15	-10.35	0.11	0.11	0.00
NSAIDs	0.25	0.25	0.15	0.25	0.26	0.02
Risk score on the index date or during the 1-year pre-index period (mean)						
CCI total score†	4.17	4.61	-17.69	4.17	4.18	0.01
$CHADS_2$ total score†	1.70	1.89	-15.70	1.70	1.69	0.01
CHA_2DS_2-VASc total score†	2.98	3.31	-20.34	2.98	2.97	0.01
HAS-BLED total score†	2.27	2.50	-19.70	2.27	2.26	0.00

Region is US census region: Northeast, Midwest, South, West, or Missing.

CCI, Charlson comorbidity index; CHF, congestive heart failure; COPD, chronic objective pulmonary disease; GERD, Gastroesophageal reflux disease; HIV, human immunodeficiency virus; NSAID, non-steroidal anti-inflammatory drug; P, proportion; STD, standardized difference; TIA, transient ischemic attack.

Table 6S. Reasons for discontinuation of medication.

	Dabigatran versus Rivaroxaban		Dabigatran versus Apixaban	
**Cohort post PSM**	**12 763**	**12 763**	**4802**	**4802**
Completed to end of study, n (%)[a]	2083 (16.3)	4252 (33.3)	1207 (25.1)	2377 (49.5)
Discontinued, n (%)[a]				
Index DOAC discontinued[b]	7079 (55.5)	5844 (45.8)	2423 (50.5)	1788 (37.2)
Therapeutic switch	2255 (17.7)	1172 (9.2)	812 (16.9)	246 (5.1)
Index DOAC dose change	1108 (8.7)	1267 (9.9)	292 (6.1)	322 (6.7)
Disenrollment from coverage	11 (0.1)	29 (0.2)	1 (0.0)	10 (0.2)
Death	227 (1.8)	199 (1.6)	67 (1.4)	59 (1.2)

Dabigatran versus rivaroxaban (July 1, 2011 and June 30, 2016); dabigatran versus apixaban (December 28, 2012 to June 30, 2016).

DOACs, direct oral anticoagulants.

[a]Percent of propensity-score matched cohort (that is, n=12 763 or 4801).
[b]Index exposure was considered discontinued if there was a treatment gap longer than the 30-day allowable gap specified from the end of the calculated days supplied
